# Giant room temperature compression and bending in ferroelectric oxide pillars

**DOI:** 10.1038/s41467-022-27952-2

**Published:** 2022-01-17

**Authors:** Ying Liu, Xiangyuan Cui, Ranming Niu, Shujun Zhang, Xiaozhou Liao, Scott D. Moss, Peter Finkel, Magnus Garbrecht, Simon P. Ringer, Julie M. Cairney

**Affiliations:** 1 School of Aerospace, Mechanical & Mechatronic Engineering, The University of Sydney, Sydney, NSW 2006 Australia; 2 Australian Centre for Microscopy and Microanalysis, The University of Sydney, Sydney, NSW 2006 Australia; 3 ISEM, Australian Institute of Innovative Materials, University of Wollongong, Wollongong, NSW 2500 Australia; 4 Aerospace Division, Defence Science and Technology Group, Melbourne, VIC 3207 Australia; 5 US Naval Research Laboratory, Washington DC, 20375 USA

**Keywords:** Electronic devices, Sensors and biosensors

## Abstract

Plastic deformation in ceramic materials is normally only observed in nanometre-sized samples. However, we have observed high levels of plasticity (>50% plastic strain) and excellent elasticity (6% elastic strain) in perovskite oxide Pb(In_1/2_Nb_1/2_)O_3_-Pb(Mg_1/3_Nb_2/3_)O_3_-PbTiO_3_, under compression along <100>_pc_ pillars up to 2.1 μm in diameter. The extent of this deformation is much higher than has previously been reported for ceramic materials, and the sample size at which plasticity is observed is almost an order of magnitude larger. Bending tests also revealed over 8% flexural strain. Plastic deformation occurred by slip along {110} <1$\bar{1}$0 > . Calculations indicate that the resulting strain gradients will give rise to giant flexoelectric polarization. First principles models predict that a high concentration of oxygen vacancies weaken the covalent/ionic bonds, giving rise to the unexpected plasticity. Mechanical testing on oxygen vacancies-rich Mn-doped Pb(In_1/2_Nb_1/2_)O_3_-Pb(Mg_1/3_Nb_2/3_)O_3_-PbTiO_3_ confirmed this prediction. These findings will facilitate the design of plastic ceramic materials and the development of flexoelectric-based nano-electromechanical systems.

## Abstract (web summary)

In materials science, it is a well-accepted fact that metals are generally ductile, and ceramics are always brittle. Here, the authors observe high level of plasticity and excellent elasticity in perovskite oxide under compression and bending with diameter of 2.1 μm.

## Introduction

Conventional wisdom dictates that most metals are ductile and almost all ceramics are brittle. The plasticity of metals is related to their atomic bonding. Valence electrons are not bound to a specific atom and there is little charge resistance during dislocation slip^1^. For ceramics, the directional covalent or/and ionic bonds restrict slip due to electrostatic repulsion, resulting in brittle fracture with only limited strain (usually less than 0.2%)^1^. In many cases, the brittle nature of ceramics limits their application, and improvements to the brittle properties of ceramics materials have been sought for decades^2^.

There are some rare exceptions to this rule. Crystals with the rock salt structure show limited plasticity due to their unique structure (slip occurs on {110} planes and along <1$\bar{1}$0> directions, where it does not bring similarly charged atoms together)^3^. Among perovskite oxides, SrTiO_3_ (STO) has been reported to display around 7% plastic deformation under uniaxial compression at a low strain rate (10^−4^)^4^. More recently, good plasticity was reported in semiconductor α-Ag_2_S and InSe single crystals^5,6^. In α-Ag_2_S, excellent plasticity was attributed to planes with weak atomic interactions and irregularly distributed sulfur–silver and silver–silver bonds^5^, while in InSe, the plasticity is thought to result from long-range In-Se Coulomb interactions across the van der Waals gap and soft intralayer In-Se bonding^6^. Flash-sintered TiO_2_ has been compressed to ~10% strain, attributed to a high density of stacking faults, nanotwins and dislocations^7^. Plastic deformation observed in nanopillars, nanowires, etc., is mostly attributed to the low chance of smaller samples containing flaws, allowing the materials’ intrinsic plasticity to be observed^8–14^.

The existence of deformable ceramics has striking potential, but systems that display this characteristic must be identified and plasticity mechanisms need to be understood in order to guide the design of such materials. Because plastic deformation is not typical of ceramics, the applications have not yet been fully considered. It is expected such properties might enable applications such as sensors or even bendable and foldable electronics^15^ where flexible ceramic film capacitors are required^16^.

Excellent elastic properties are especially desirable^17^ for functional oxides. A mechanical bending moment enables a dielectric material to polarize, giving rise to flexoelectricity. Flexoelectricity has a strong scaling effect and is therefore significant at micro/nanoscales. For this reason, it has the potential to be used for electromechanical actuators and sensors that can be integrated into advanced nano-/microelectromechanical systems (N/MEMS)^18,19^, meeting the requirement for the millions of micro- and nanoscale sensors to be employed during the expected rapid implementation of the Internet of Things.

Perovskite oxides are of great interest to both geophysics and materials science^20^. In geophysics, a MgSiO_3_-rich perovskite phase is thought to account for 50−90% of the volume of the region of the earth that controls seismic activity^21,22^ (i.e., the 670 km seismic discontinuity to the core-mantle boundary^19^). In the field of materials science, perovskites are of interest because they exhibit useful flexoelectric, dielectric, piezoelectric, ferroelectric, ferromagnetic, multiferroic, superconducting, and photovoltaic properties, as well as colossal magnetoresistance^23^. Pb(In_1/2_Nb_1/2_)O_3_-Pb(Mg_1/3_Nb_2/3_)O_3_-PbTiO_3_ (PIN-PMN-PT) is a ternary relaxor ferroelectric perovskite. Single crystal PIN-PMN-PT exhibits outstanding flexoelectric, piezoelectric, and electromechanical properties (flexoelectric coupling coefficient *µ*_12_ of 5 × 10^4^ nC∙m^−1^, piezoelectric coefficient of *d*_33_ ~ 2000 pC/N and electromechanical coupling factor of *k*_33_ ~ 90%) compared to traditional Pb(Zr,Ti)O_3_ piezoelectric ceramics (*d*_33_ < 500 pC/N, *k*_33_ < 75%)^24–26^. These extraordinary electromechanical coupling functionalities mean that the mechanical properties are of great interest.

## Results

PIN-PMN-PT samples were first characterised by transmission electron microscopy (TEM). Even prior to deformation experiments, clues to the potential plastic behaviour of PIN-PMN-PT were already apparent. During the preparation of thin specimens for TEM, it was noted that the edges of ~3 µm tripod-polished samples were not flat (Supplementary Fig. 1a, b), and a high density of entangled dislocations (Supplementary Fig. 1) was present in the resulting TEM samples. Compression, tensile and bending tests on PIN-PMN-PT were carried out by using a combination of TEM, scanning electron microscopy (SEM) and nanomechanical test systems. The experimental setup is shown in Supplementary Fig. 2.

**Fig. 1:**
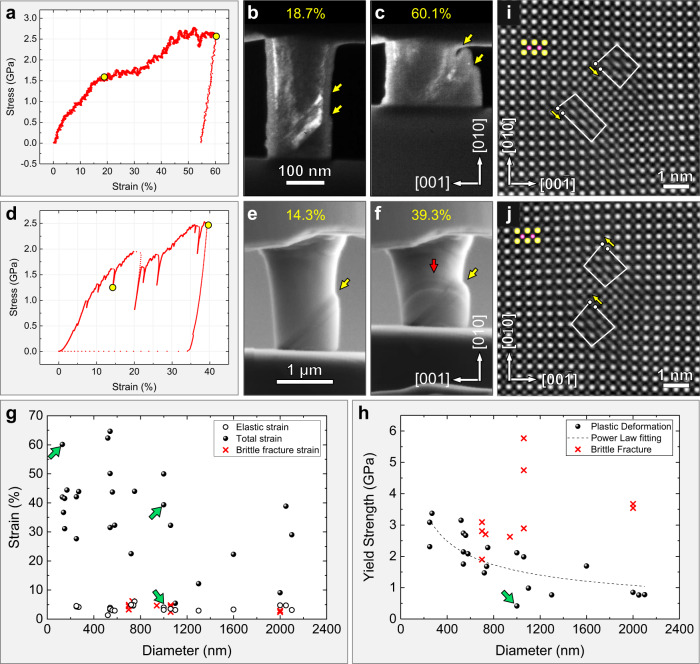
Compression tests of sub-micro and micrometre scale pillars. **a** An engineering stress–strain curve acquired during the compression of 140 nm diameter pillar, with a loading direction along [010]. **b**–**c** Snapshots from a real-time video recording of a compression test, at strains of 18.7% and 60.1%, respectively (labelled as yellow circles in **a**). Slip bands along (011) crystallographic plane and [01$\bar{1}$] direction is indicated by yellow arrows. Here, both slip plane and slip direction are determined from the change of contrast in TEM images. **d** An engineering stress–strain curve from a compression test of a 1 µm pillar. **e**–**f** Video snapshots corresponding to strains of 14.3% and 39.3% (yellow circles in **d**). Two slip bands (oriented (110)[01$\bar{1}$] and (1$\bar{1}$0)[110]) are indicated by yellow and red arrows. **g** Strain as a function of pillar diameter, showing plastic strain (hollow circles) and total strain (spheres) for plastic-deformed pillars and fracture strain (red crosses) for brittle-fracture samples. **h** Yield strength as a function of pillar diameter, showing yield strength for plastic-deformed pillars (spheres) and brittle-fracture pillars (red crosses). Dashed black curve: fitted yield strength–diameter curve for plastic-deformed pillars, with a function of *y* = 56.9*x*^−0.52^. Green arrows indicate the strain/stress value corresponding to the pillars shown in Fig. 1**a–c** and **d**–**f**. **i**–**j** STEM-HAADF images showing pairs of partial dislocations with Burgers vectors of $\frac{1}{2}$*a*[011] (**i**) and $\frac{1}{2}$*a*[0$\bar{1}\bar{1}$] (**j**). The partial dislocations are separated by stacking faults.

Because plasticity has previously been observed in nanoscale ceramics during compression, we first tested the properties of our PIN-PMN-PT by preparing round pillars with diameters from 130 nm to 270 nm for compression experiments in a TEM. Results are shown in Fig. 1a–c and Supplementary Figs. 3 and 4. Figure 1a shows an engineering stress–strain curve from a 140 nm diameter pillar. The slope of the curve starts to decrease from ~5% strain. Two short stress plateaus appear when the strain reaches ~15% and ~44% respectively, typical of plastic deformation. The total compression strain of the pillar exceeds 60%, over 50% of which is plastic. This giant strain far surpasses the expected deformability of ceramic materials^27^ and is much higher than has been previously reported in micro/nanopillars^9,10,13^. Snapshots captured from a video of the compression are shown in Fig. 1b–d. Slip bands (indicated by yellow arrows) develop on the (011) crystallographic plane, along the [01$\bar{1}$] direction. Similar phenomena were observed for the other eight pillars with diameters ranging from 130 ~ 270 nm (Supplementary Figs. 3 and 4).

As mentioned earlier, previous studies showed that STO single crystals displayed surprisingly high plasticity, with a plastic strain of ~7%^4,28^. Here, we compare the compression behaviour of PIN-PMN-PT and STO by also compressing single crystal STO pillars along the same orientation (Supplementary Figs. 5 and 6). Five out of six STO pillars underwent brittle fracture. The smallest pillar, at 150 nm, was the only one that did not fracture, suggesting that STO undergoes a brittle-to-plastic transition with a critical pillar diameter of around 150 ~ 180 nm, while PIN-PMN-PT has significantly better plasticity (all PIN-PMN-PT pillars show plastic deformation). The maximum observed plastic strain was 17.8% for an STO pillar with the diameter of 180 nm, where PIN-PMN-PT pillars with a similar diameter typically displayed >40% strain.

To determine the effect of pillar size on the deformation behaviour in PIN-PMN-PT, larger pillars were fabricated with diameters ranging from 500 nm to 2.1 µm. Most of them displayed plasticity and some were brittle. An engineering stress–strain curve of a 1 µm diameter pillar is provided in Fig. 1d–f. Strain bursts were observed, characterized by serrated yielding in the stress–strain curve. Similar rapid bursts of deformation are typical of tests conducted on micrometer-scale metal pillars^29^. Video snapshots in Fig. 1e–f correspond to strain of 14.3% and 39.3%, respectively. Slip initiates along the (011) plane and [01$\bar{1}$] direction, as indicated by the yellow arrow in Fig. 1e. With further deformation, another slip band (110) [$\bar{1}$10] is activated, indicated by the red arrow in Fig. 1f and deformation proceeds until the strain reaches 39.3%. Compression test results from fourteen more pillars with diameters ranging from 500 nm – 2.1 µm are shown in Supplementary Figs. 7 and 8 and another detailed example of excellent deformability for a 2.1 µm diameter pillar can be found in Supplementary Fig. 9 (($\bar{1}$10) [110] slip and 39.1% strain). About 60, 50 and 40% strain were observed in pillars of 500 nm, 1 μm and 2.1 μm diameters, respectively, as shown in Supplementary Figs. 7a, b, 8c, and 9, which far surpasses the plasticity observed in STO. Figure 1g, h summarises the results of all compression tests, where Fig. 1g shows the strain (calculated according to the pillar length where the test stops) and Fig. 1h shows the yield strength for pillars with the diameter ranging from 150 nm to 2.1 µm. All samples < 700 nm diameter underwent plastic deformation, while some larger samples were brittle (an example is shown in Supplementary Fig. 10). The yield strength is typically higher for smaller samples (Fig. 1h). These results are consistent with the literature on size effects in metals and ceramic pillars^29–34^. However, though both STO and PIN-PMN-PT are perovskite oxides, their deformability differs greatly, and the size effects kick in at a much larger size scale for PIN-PMN-PT. This suggests that the intrinsic plasticity of the PIN-PMN-PT is much greater. The one order of magnitude improvement (from 150 nm to 2.1 µm) of plasticity observed here in PIN-PMN-PT is smaller than the size effect reported in metals, in terms of strength and work hardening rate, which show effects up to 20 µm. Due to the limited load of the Hysitron PI 85 L picoindenter, the maximum diameter we tested was 2.1 µm, and the deformation behavior of PIN-PMN-PT pillars with a diameter larger than 2.1 µm is still an issue to be explored. The elastic compression strain is consistent for all samples, at an average of 3.8% and a maximum of 6.2%. The composition of PIN-PMN-PT used here is close to the morphotropic phase boundary at which an adaptive ferroelectric phase has been proposed, which can easily transform to other phases upon the mechanical strain. Our previous work shows that a reversible polydomain-rhombohedral to monodomain-orthorhombic phase transition happens under compression^35^, which is thought to contribute to the large elastic strain observed here. To better understand the plastic deformation mechanism, deformed samples were further thinned by a focused ion beam (FIB) into TEM foils. Scanning transmission electron microscopy−high-resolution high-angle annular dark-field (STEM − HAADF) images from a deformed area (Fig. 1i, j) show climb-dissociated pairs of partial dislocations with Burgers vector $\frac{1}{2}a$ <011> , separated by a stacking fault (see also Supplementary Figs. 11–13).

**Fig. 2:**
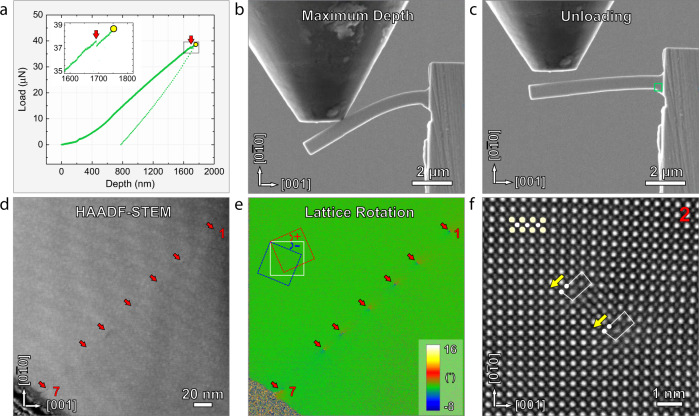
Bending test of PIN-PMN-PT. **a** A load-depth curve obtained during bending a cantilever beam along a loading direction of [010]. The inset shows the enlarged curve of the rectangular area, where an abrupt decrease in mechanical load is evident. **b** Snapshot captured from in-situ video corresponding to the maximum depth of the indenter. **c** An SEM image showing cantilever beam after unloading. Irreversible deformation can be clearly revealed by comparison with Supplementary Fig. 2g. **d**–**e** A STEM-HAADF image and the corresponding GPA analysis of lattice rotation, with dislocations indicated by red arrows. Dislocations #1 and #7 are labelled. The lattice rotation angle is indicated by the color bar. **f** High-resolution STEM-HAADF images showing dislocation #2, which includes a pair of partial dislocations with Burgers vector of $\frac{1}{2}$*a*[01$\bar{1}$].

Dog-bone pillars and cantilever beams were fabricated for tensile and bending tests. The experimental setup is described in Supplementary Fig. 2d–g. Tensile tests (Supplementary Fig. 14) of a dog bone sample of dimensions 1.9 µm × 0.5 µm × 0.1 µm revealed an elastic strain of 4.0%, but no plastic deformation. Figure 2a shows a load–displacement curve obtained from an in-situ bending test. After deformation, the cantilever shows residual plastic deformation (1.4%), consistent with an abrupt decrease in mechanical load, indicated by a red arrow in the inset curve in Fig. 2a. Figure 2b, c show video snapshots at maximum load and after unloading (an image prior to bending is shown in Supplementary Fig. 2g). A maximum flexural strain of 8.2%, where 6.8% is elastic and 1.4% is plastic (details in SI) that occurs at the root of the cantilever beam. Figure 2d is a low magnification high-resolution STEM-HAADF image taken from the area marked in green in Fig. 2c. Contrast is indicated by red arrows and numbers. Lattice rotation mapping derived from Geometric Phase Analysis (GPA, see methods) displays this contrast more clearly, Fig. 2e, highlighting dislocation cores^36,37^. Dislocations 1–6 are the same. Figure 2f is a high-resolution STEM-HAADF image of dislocation #2, which consists of a pair of partial dislocations with Burgers vector of $\frac{1}{2}$*a*[01$\bar{1}$] and a stacking fault between them, consistent with the defects observed in compressed pillars. Dislocation #7 is different (Supplementary Fig. 15b, c) and is assumed to be affected by the proximity of the surface of the cantilever beam.

For perovskite oxides, it is generally accepted that, at ambient temperature, the preferred slip system is {110} <1$\bar{1}$0 > , with *a* < 1$\bar{1}$0 > dislocations^19^. This type of dislocation is usually dissociated into two partials due to the high energy of two extra atomic planes. Previous studies on as-grown single/double crystals, polycrystals, or thin films show that *a* < 1$\bar{1}$0> dislocations are dissociated either in a glide or a climb mode^21^^,^^36–39^. Unexpectedly, we have observed climb-dissociated dislocation core structures, which would normally be expected to form at elevated temperatures because the climb is a diffusion-assisted process^40^. Instead, *a* < 110> dislocations formed during room temperature deformation might be expected to dissociate in a slip configuration^40^, as was previously reported for compression-tested KNbO_3_^41^. We note here that there might be a high density of point defects in the PIN-PMN-PT. These point defects enable the diffusion that is required to form climb-dissociated dislocations, leading to much better deformability compared to other perovskites such as STO or KNbO_3_.

In perovskites oxides, vacancies are far more common than interstitials^20^. Lead vacancies (${V}_{{Pb}}^{{\prime} {\prime} }$) and oxygen vacancies (${V}_ {O}^{\bullet \bullet }$) are the most important vacancies in lead-based perovskites, where ${V}_{{Pb}}^{{\prime} {\prime} }$ forms due to the volatility of lead at elevated temperature, or donor dopants, while ${V}_{O}^{\bullet \bullet }$ exists owing to the loss of oxygen at high temperature or acceptor dopants. The face-centred cubic lattice formed by Pb^2+^ and O^2−^ determines the dislocation and slip behavior^20^. Consequently, the existence of ${V}_{{Pb}}^{{\prime} {\prime} }$ and ${V}_{O}^{\bullet \bullet }$ in PIN-PMN-PT could considerably influence the observed plasticity. Studies of the effect of vacancies on the deformation behaviour of alloys or intermetallic compounds show varying results: vacancies may facilitate or deteriorate plasticity, depending on their type and distribution^42–44^.

**Fig. 3:**
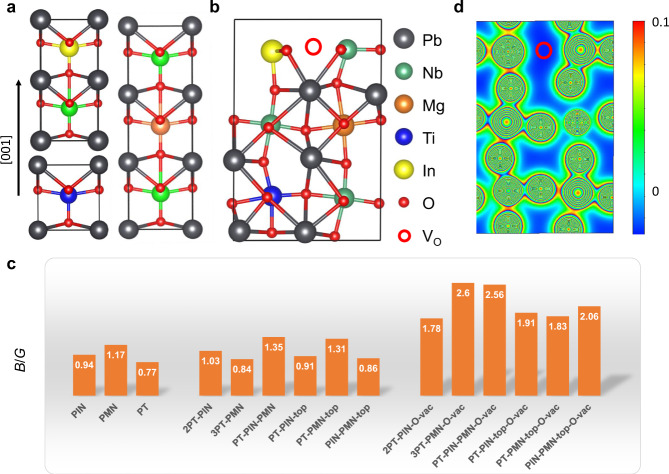
First principles atomistic investigation for the plasticity in PIN-PMN-PT. **a** Three sub-unit cells for Pb(In_1/2_Nb_1/2_)O_3_ (PIN), Pb(Mg_1/3_Nb_2/3_)O_3_ (PMN) and PbTiO_3_ (PT). **b** An example of relaxed atomic structure containing interfaces formed by one PIN, one PMN and one PT with one oxygen vacancy (${V}_{O}$) at the interface of PIN and PMN. **c** Calculated bulk modulus/shear modulus (*B*/*G*) ratios for various bulk, pristine interfaces, and interfaces with ${V}_{O}$. Higher *B*/*G* ratios (>1.75) suggest ductile behaviour in PIN-PMN-PT. **d** Calculated valence charge density 2D contour plot (colours assigned recursively) on the (020) plane of the structure shown in **b**. The strength of covalent bonding is indicated by the colour bar. The presence of ${V}_{O}$ eliminates the local covalent bonds.

To trace the possible microscopic origin of the observed excellent plasticity, we conducted first-principles atomistic simulations based on density functional theory (DFT). The results are given in Fig. 3. On the basis of a simplified model, PIN-PMN-PT is composed of three sets of subunits, PIN, PMN and PT (Fig. 3a). Atomic-scale Energy-Dispersive X-ray Spectroscopy (EDS) mapping (Supplementary Fig. 16) indicates that the cations are uniformly distributed at the atomic level, suggesting a high density of mini-interfaces between the three subunits. Relaxed atomic structure and lattice constants of the bulk and interfaces are shown in Supplementary Figs. 17 and 18, and Supplementary Tables 1 and 2. Calculated interface formation energies (shown in Supplementary Fig. 19) suggest that the presence of interfaces promote the concentration of *V*_*O*_ but not ${V}_{Pb}$. Favourable *V*_*O*_ sites in different side-by-side and top-down interface systems are shown in Supplementary Fig. 20. Interestingly, these calculations show that it is energetically favourable to form oxygen vacancies (but not lead vacancies) at these interfaces to mitigate the large lattice mismatch (Supplementary Fig. 21). That is, the three subunits that make up the PIN-PMN-PT naturally facilitate a uniformly distributed high density of *V*_*O*_. As an example, the atomic structure of 1PIN-1PMN-1PT containing one oxygen vacancy is shown in Fig. 3b. To assess the corresponding ductility, we calculated the elastic constants and derived the bulk modulus (*B*)^45^ and the anisotropic shear modulus (*G*) on the (110) plane along <1$\bar{1}$0> direction for different single tetragonal crystalline species^46^, as shown in Fig. 3c and Supplementary Table 3. The Pugh’s *B*/*G* ratio is widely used to index ductility, with a critical value of 1.75 indicating a transition from brittle to ductile behaviour^43,44^. For bulk PIN, PMN, and PT, and their pristine interfaces, the calculated *B*/*G* ratios are well below 1.75 (hence brittle). By contrast, the *B*/*G* ratios for interfaces containing *V*_*O*_ are systematically enhanced, most well above 1.75 (hence ductile). Valence charge density analysis reveals that the presence of *V*_*O*_ can dramatically weaken the covalent bonding (see Supplementary Figs. 3d and 22). For comparison, ${V}_{{Pb}}$ actually deteriorates the ductility. Thus, based on the DFT results, we attribute the excellent plasticity of PIN-PMN-PT to the high density of *V*_*O*_ at the PIN/PMN/PT interfaces (see Supplementary Fig. 20).

**Fig. 4:**
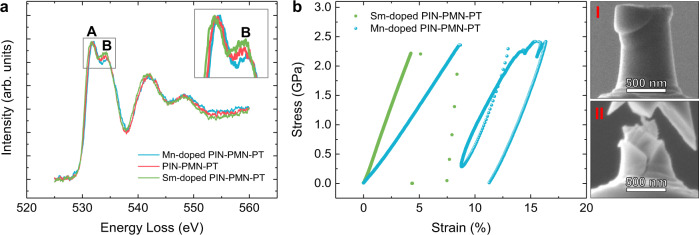
Analysis of the origin of excellent plasticity. **a** O-EELS obtained from Mn- (blue), Sm- (green) doped and undoped (red) PIN-PMN-PT. The enlarged image (inset) shows the intensity difference of peak B for Mn-, Sm- doped and undoped PIN-PMN-PT, where a lower intensity suggests a higher ${V}_{O}^{\bullet \bullet}$ concentration. **b** Engineering stress ‒ strain curves obtained from in-situ compression tests of Mn- (blue sphere) and Sm- (green circle) doped PIN-PMN-PT. Images I and II show SEM images of the compressed Mn-doped and Sm-doped PIN-PMN-PT respectively.

On the basis of DFT predictions, we investigated the ${V}_{O}^{\bullet \bullet }$ levels and mechanical behaviour of PIN-PMN-PT crystals that are expected to be ${V}_{{Pb}}^{{\prime} {\prime} }$-rich and ${V}_{O}^{\bullet \bullet }$-rich, (Sm-doped^47^ and Mn-doped^26^ crystals respectively), and compared them to the original undoped PIN-PMN-PT crystal. Electron energy-loss spectra (EELS) of O were collected to verify the existence of oxygen vacancies, shown in Fig. 4a. A lower intensity is observed for the O-k edge fine structure peak B compared to A for all three EELS curves. It is known that the peak at position B being lower than the peak at position A is an indication of oxygen deficiency in perovskite oxides^48–50^, suggesting that ${V}_{O}^{\bullet \bullet }$ with appreciable concentrations exist in all three samples. Furthermore, the inset image shows that peak B is larger for the Sm-doped sample than the undoped crystal, indicating a lower ${V}_{O}^{\bullet \bullet }$ concentration, and is smaller for the Mn-doped sample, indicating a higher ${V}_{O}^{\bullet \bullet }$ fraction.

According to the DFT predictions, the ${V}_{O}^{\bullet \bullet }$-rich (Mn-doped^26^) samples are more likely to be ductile and the ${V}_{{Pb}}^{{\prime} {\prime} }$-rich, (Sm-doped^47^) crystals are more likely to be brittle. Compression tests were performed on both samples. Example engineering stress–strain curves and SEM images of compressed pillars are shown in Fig. 4b (details in Supplementary Figs. 23 and 24). Six ~600 nm diameter pillars were fabricated for each sample type. All Mn-doped PIN-PMN-PT pillars showed plasticity, while half of the Sm-doped PIN-PMN-PT pillars underwent brittle fracture, indicating that the Mn-doped sample had superior plasticity. In the examples shown in Fig. 4b, the Sm-doped sample has fractured in a brittle way, while the Mn-doped sample has slip bands on the pillar and a stress plateau and strain burst on the stress–strain curve. The results of this comparison experiment are consistent with our hypothesis of ${V}_{O}^{\bullet \bullet }$-induced plasticity. In addition to doping, vacuum annealing is another way to introduce oxygen vacancies. It can be expected that vacuum annealing and Mn-doping would act synergistically to improve the plasticity of PIN-PMN-PT.

## Discussion

It has been proposed by Zubko et al. that dislocations contribute significantly to flexoelectricity in STO^51^. Tang et al. and Gao et al. measured the strain gradient around dislocations by extracting Bi/Sr positions from STEM-HAADF images and calculating the flexoelectric polarization in multiferroic BiFeO_3_ and paraelectric STO^37,52^, which was found to be several µC cm^−2^. The flexoelectric effect is expected to be large, because relaxor ferroelectric PIN-PMN-PT shows outstanding flexoelectricity compared to other perovskite oxides. Take STO as an example, the flexoelectric coefficient *µ*_12_ is about 7 nC m^−1^ ^24,25,51^, while that of PIN-PMN-PT is about 5.0 × 10^4^ nC m^−1^ ^24^, a difference of four orders of magnitude.

Here, in order to measure the flexoelectric polarization around a pair of partial dislocations (introduced by plastic deformation), we extracted Pb atom positions from STEM-HAADF images firstly (details in SI) and calculated the maximum strain gradient (∇*S*) to be about 3.5 × 10^9^ m^−1^ ([0$\bar{1}$1] lattice strain gradient along the [0$\bar{1}\bar{1}$] direction), which is 3 times that reported by Gao et al. around [010] dislocations in a STO bicrystal^52^. Supposing the flexoelectric coefficient of PIN-PMN-PT [110] is comparable to that of *µ*_12_^24^, the local flexoelectric polarization (1~2 unit-cells) around dislocations is estimated to be about 10^7^ µC·cm^−2^ according to *P*_*f*_ = *u* × ∇*S*, where *P*_*f*_ is flexoelectric polarization, *u* is flexoelectric coefficient, and ∇*S* is the gradient of the horizontal lattice constant along the vertical direction. However, this large calculated polarization is thought to be an overestimate for two reasons. 1) In the case of such high strain gradients, higher-order coupling terms of flexoelectric polarization and strain gradient, which is nonlinear, should not be neglected, and the magnitude of those terms is still unclear. 2) For smaller samples, permittivity (*ɛ*) is expected to decrease as a result of a size effect^53^, and the flexoelectric coefficient *µ*, which is a function of *ɛ* in a manner of *µ* = *f* ∙ *ɛ*, should also be smaller than the corresponding bulk value (here *f* is flexo-coupling coefficient, about 10 V for PTO-based relaxor ferroelectrics). However, this large polarization should give rise to a large number of bound charges. To screen these bound charges, free charges will accumulate. Transport properties or even magnetic properties around these dislocations can also be affected due to free charges. For slip bands, where a strain gradient also exists (as shown in Supplementary Fig. 11e), the situation would be similar. As the strain gradient around a slip band is much smaller than it is around dislocations, the flexoelectric effect will be smaller. The movement of dislocations and the introduced slip bands make a functional region, which is potentially applicable for flexoelectric-based micro- and nanoscale electronic devices.

In addition to strain gradients around dislocations and slip bands, bending-induced elastic strain gradients are also of great interest for flexoelectricity because of their reversibility. The maximum elastic strain introduced by the bending test is calculated to be 6.8% at the root of the cantilever beam, and the width (*b*) of the cantilever beam is 0.67 µm, which gives rise to a strain gradient of about 2 × 10^5^ m^−1^ $(\nabla S=\frac{6.8 \% }{0.335\,\mu {{{{{\rm{m}}}}}}}\approx 2\times {10}^{5}{{{{{{\rm{m}}}}}}}^{-1})$. Flexoelectric polarization from the elastic bending strain gradient is estimated to be about 1 × 10^3^ µC⋅cm^−2^. Here strain gradient ∇*S* is the horizontal ([001]) lattice strain gradient along the vertical direction ([010]). The calculated flexoelectric polarization is 1 ~ 2 orders of magnitude larger than the ferroelectric polarization of known ferroelectrics. For example, the ferroelectric polarization of PbTiO_3_ is about 75 µC⋅cm^−2^, the ferroelectric polarization of BiFeO_3_ is around 90 µC⋅cm^−2^, and the polarization of BaTiO_3_ is about 26 µC·cm^−2^ ^54–56^. The calculated flexoelectric polarization is also 4 ~ 5 times that of the recently-reported ferroelectric polarization of super-tetragonal PbTiO_3_^57^. An even larger flexoelectric polarization would be expected if a lower strain rate is used, according to Deng’s work^58^. This large flexoelectric polarization is also likely to be an overestimate for reasons mentioned above. However, even if the real flexoelectric polarization is 1/10 of the calculated 1 × 10^3^ µC⋅cm^−2^, it is still large enough (100 µC⋅cm^−2^) to switch the local ferroelectric polarization, and to be used in flexoelectric-based sensors.

The excellent deformability in ${V}_{O}^{\bullet \bullet }$-rich PIN-PMN-PT is particularly promising for flexoelectric-based sensors, because it was reported that the effective flexoelectricity of oxygen-depleted perovskite oxide is two orders of magnitude larger than for a stoichiometric sample^59^. Combined with the scaling effects of flexoelectricity and the super large flexoelectric coefficient of PIN-PMN-PT, these provide exciting opportunities for high-performance flexoelectric-based N/MEMS devices.

Recent work by Höfling et al. shows that mechanical dislocation imprinting improves the dielectric and piezoelectric properties by a few orders of magnitude^60^. It makes dislocations a potential interest. Deformation is a common way to generate dislocations. Improving plastic deformability is crucial for investigating the dislocation effect on functionalities. The oxygen vacancies assisted plastic deformation identified here do help in that.

To summarize, we have revealed excellent deformability in relaxor ferroelectric PIN-PMN-PT micron/submicron single crystals pillars. A maximum elastic strain of >6% and plastic strain >50% were observed during compression tests, while a flexural strain of 8.2% was achieved for a bent cantilever beam. Pairs of $\frac{1}{2}\,$*a* < 011> climb-dissociated partial dislocations accommodate the plastic deformation. Based on first-principles calculations, confirmed by experiments, we propose that the observed excellent plasticity is attributed to not only a decrease in the specimen size but also a high ${V}_{O}^{\bullet \bullet }$ concentration. This suggests that it might be possible to alter the plasticity of ceramic materials by deliberate engineering of point defects, which paves the way towards the design of ductile ceramics, and implies that more attention should be paid to the previously ignored mechanical properties of functional oxides. The giant strain gradient generated by elastic bending and dislocations gives rise to considerable flexoelectric polarization, which can be used in sensors. These results will facilitate the development of flexoelectric-based flexible electronic devices and N/MEMS.

## Methods

### Materials

The experimental work reported in this paper was performed using [011] poled PIN-PMN-PT single crystal plates (CTS Advanced Materials, with nominal composition 0.24PIN-0.44PMN-0.32PT, grown via the modified Bridgeman method) with MPB composition, a relative permittivity of 4000, dimensions of 12 × 4 × 4 mm^3^, and the surface polished to 50–110 nm. Sm-doped and Mn-doped PIN-PMN-PT (TRS Technologies) single crystals were grown by a modified Bridgeman method and STO is commercial single crystal.

### Sample preparation

#### Micro-pillars preparation for compression, tensile and bending tests

The PIN-PMN-PT single crystal was first cut into slices of 0.5 mm in thickness, then further thinned using tripod polishing to ~500 nm at the front edge. Pillars used for in-situ tests were fabricated at the thin edge by using FIB. Columnar pillars with an aspect ratio (height/diameter) of 2:1 ~ 3:1 were prepared for compression tests. The FIB was operated at 30 kV using a current of 1 nA for coarse milling and 5 pA ~ 300 pA for final milling of pillars with diameters ranging from 130 nm ~ 2.1 µm. The pillar taper angles are estimated to be around 3°. The diameter of the top surface was used for stress calculation, which is the first part of the sample to undergo plastic deformation. Cantilever beams for bending tests were prepared with FIB operating at 30 kV and using a current of 50 pA for final milling. The length, width and depth are 6.5, 0.67 and 0.8 µm, respectively. Dog bone-shaped pillars were prepared for tensile tests, and 30 kV, 5 pA were used for final milling.

#### TEM sample preparation

The deformed pillars were lifted-out using a tungsten manipulator onto a copper base, and then thinned to electron transparency (~ 50 nm) for TEM observation. 10 kV and 10 pA were used for FIB final milling. 5 kV, 10 pA and 2 kV, 10 pA were used for the final cleaning of the surface. To protect the pillars from FIB damage, platinum was deposited around the pillars before thinning.

#### TEM sample preparation for O-K EELS

TEM samples for O-K EELS were prepared by grinding using tripod polisher and ion milling employing a Gatan precision ion polishing system II (PIPS II). 4° and 0.5 kV were used for final milling.

### In-situ mechanical tests

In-situ compression experiments were carried out in both a TEM (JEOL JEM 2100) and an SEM (Zeiss Ultra), while in-situ tensile and bending tests were conducted in the SEM. The JEOL JEM 2100 uses a high-brightness LaB_6_ electron source. It is equipped with Xarosa (4 k × 4 k) as well as Veleta Ultrascan (2 k × 2 k) cameras. In the TEM, in-situ compression tests of pillars with diameters around 200 nm were carried out by using a Hysitron PI 95 Picoindenter with a flat diamond tip. As the load applied is limited to 1.5 mN for the PI 95 Picoindenter, the requirement for thin sample in the TEM, we carried out the in-situ compression experiment of the larger pillars by using a Hysitron PI 85 L picoindenter inside an SEM, with a specially designed system for applying loads up to 10 mN. This system allows real-time observation of deformation process (i.e. slip band development, slip planes and slip directions). The load was applied to pillars by moving the indenter toward the pillars in the displacement control mode. The displacement rates were 1 nm⋅s^−1^ and 2 nm⋅s^−1^ for compression of pillars of around 200 nm in diameter and from 500 nm ~ 2.1 µm in diameter, respectively. For the tensile test, a displacement rate of 1 nm⋅s^−1^ was used. For the bending test, a higher displacement rate − 4 nm⋅s^−1^ was used.

### Microstructure investigation of the deformed pillars

A JEOL JEM 2100 TEM and a FEI Themis-Z Double-corrected 60-300 kV S/TEM were used to observe the compressed pillars. High-resolution STEM-HAADF images, EDS element mapping and O-K edge EELS were acquired using the FEI Themis-Z S/TEM. The convergence and collection angle under the STEM-HAADF mode are 17.9 mrad and 50–200 mrad, respectively. The strain was analysed using free Geometric Phase Analysis script (by C.T. Koch)^61^. EELS of the O-K edge was acquired under the TEM mode at a collection angle of 100 mrad. Dual-EELS was used and zero peak was corrected for all three samples. The energy resolution is estimated to be 1.0 eV, measured from the full width at half maximus of zero loss peak, while an energy dispersion of 0.025 eV/ch was employed. The point resolution of Themis-Z under the STEM mode is around 0.6 Å (operated at 300 kV). It is equipped with X-FEG high-brightness gun, Monochromator, ChemiSTEM (Super-X) EDS detectors as well as a Gatan Quantum ER/965 GIF ( < 0.14 eV (1 s)) with Dual-EELS.

### First-principles simulation

DFT calculations were performed using the plane-wave pseudopotential total energy method as implemented in the VASP code^62,63^. Projector augmented wave potentials^64^ and the generalized gradient approximation^65^ were used for exchange–correlation. A plane-wave basis set was used with an energy cut-off of 500 eV. The summation over the Brillouin zone for the bulk structures was performed on a ~0.06 Å^−1^ spacing Monkhorst–Pack **k**-point mesh for all calculations. For all systems, atomic relaxation was allowed until all the forces were less than 0.01 eV/Å. For charge density calculations, Pb-5*d*, Nb-4*p*, Mg-2*p*, Ti-3*p*, and In-4*d* semi-core states were treated as valence states to ensure high accuracy. Additional computational details can be found in the Supporting Information.

## Supplementary information

Supplementary Information

## Data Availability

The authors declare that the data supporting the findings of this study are available within this paper and its supplementary information files.
