# Supplementary material for: Giant room temperature compression and bending in ferroelectric oxide pillars
Source: Nat Commun. 2022 Jan 17;13:335. doi: 10.1038/s41467-022-27952-2 (PMC8764079; doi:10.1038/s41467-022-27952-2)
Supplement: Supplementary file 1 — Supplementary Information [file 41467_2022_27952_MOESM1_ESM.pdf]

Supplementary information for

Giant Room Temperature Compression and bending Deformability in Ferroelectric Oxide Pillars

Ying Liu[1,2], Xiangyuan Cui[1,2], Ranming Niu[1], Shujun Zhang[3], Xiaozhou Liao[1], Scott D. Moss[4], Peter Finkel[5], Magnus Garbrecht[2], Simon P. Ringer[1], Julie M. Cairney[1,2*]

[1]School of Aerospace, Mechanical & Mechatronic Engineering, The University of Sydney, NSW 2006, Australia;
[2]Australian Centre for Microscopy and Microanalysis, The University of Sydney, NSW 2006, Australia;
[3]ISEM, Australian Institute of Innovative Materials, University of Wollongong, NSW 2500, Australia;
[4]Aerospace Division, Defence Science and Technology Group, VIC 3207, Australia;
[5]US Naval Research Laboratory, Washington DC, 20375, USA

This PDF file includes:

1. Bent TEM specimens;
2. Experimental set up of compression, tensile and bending tests;
3. Compression test results of PIN-PMN-PT pillars with diameters ranging from 130 nm ~ 270 nm, with a loading direction of $[010]_{pc}$;
4. Compression test results of $SrTiO_3$ (STO) single crystal pillars with diameter ranging from 150 nm to 260 nm, and loaded along [010];
5. Compression of PIN-PMN-PT pillars with diameters ranging from 500 nm ~ 2.1 μm and loading direction along $[010]_{pc}$:
6. Plastic instability of large PIN-PMN-PT;
7. Microstructure analysis of compressed pillars;
8. Tensile test results of PIN-PMN-PT with loading direction along $[010]_{pc}$;
9. Bending test results of PIN-PMN-PT along $[010]_{pc}$;
10. Atomic scale EDS mapping of PIN-PMN-PT;
11. Three sets of tetragonal subunits;
12. Theoretical microstructural model contains multiple mini-interfaces in the PIN-PMN-PT sample;
13. Oxygen vacancies in PIN-PMN-PT;
14. Mechanical properties from first principles;
15. Electronic structure: bonding analysis using charge density plots;
16. Comparison of the mechanical test results of Mn-doped ($V_O^{\cdot\cdot}$-rich) and Sm-doped (V_{Pb}''-rich) PIN-PMN-PT.

1. Bent TEM specimens:

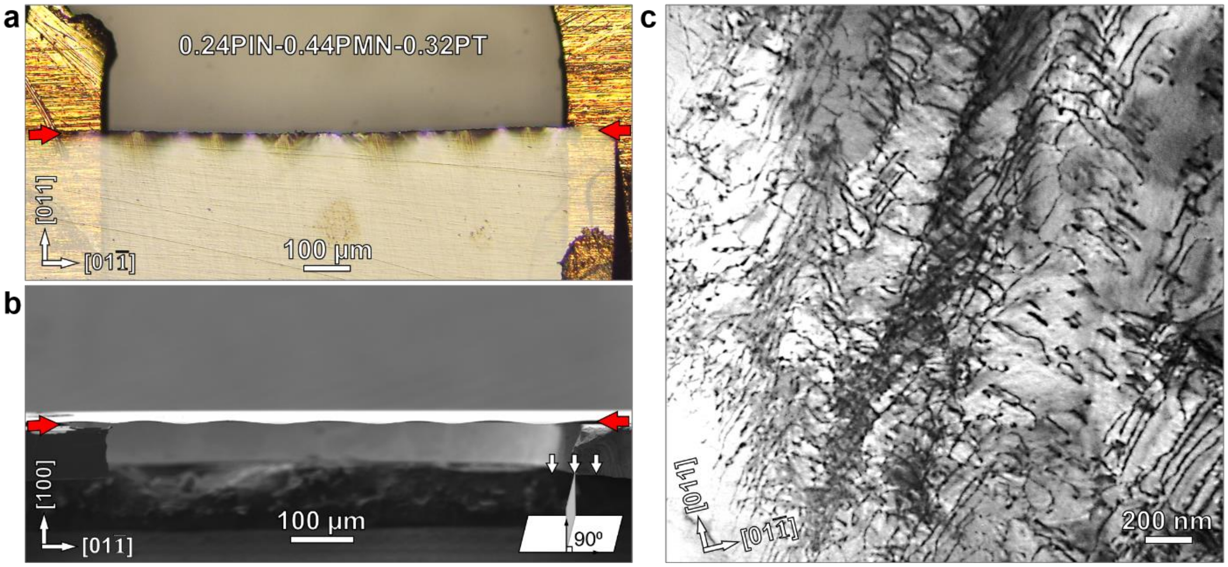

Supplementary Fig. 1. Deformation at the edge of a PIN-PMN-PT thin flake and a corresponding high dislocation density. a – **b**, Optical microscopy and scanning electron microscopy (SEM) images of a wedge-shaped PIN-PMN-PT flake prepared by tripod polishing. The thinnest edge of the flake is around 500 nm. **a**, Bright and dark contrasts are induced by the focus difference at the edge of the flake (i.e. the bright and dark areas are not at the same height). **b**, An SEM image of the edge view, taken along $[011]_{pc}$ ("pc" means pseudo cubic here) confirms this effect; the wavy edge is indicated by a pair of opposite arrows. **c**, A bright-field TEM image shows a high density of entangled dislocations.

2. Experimental set up of compression, tensile and bending tests:

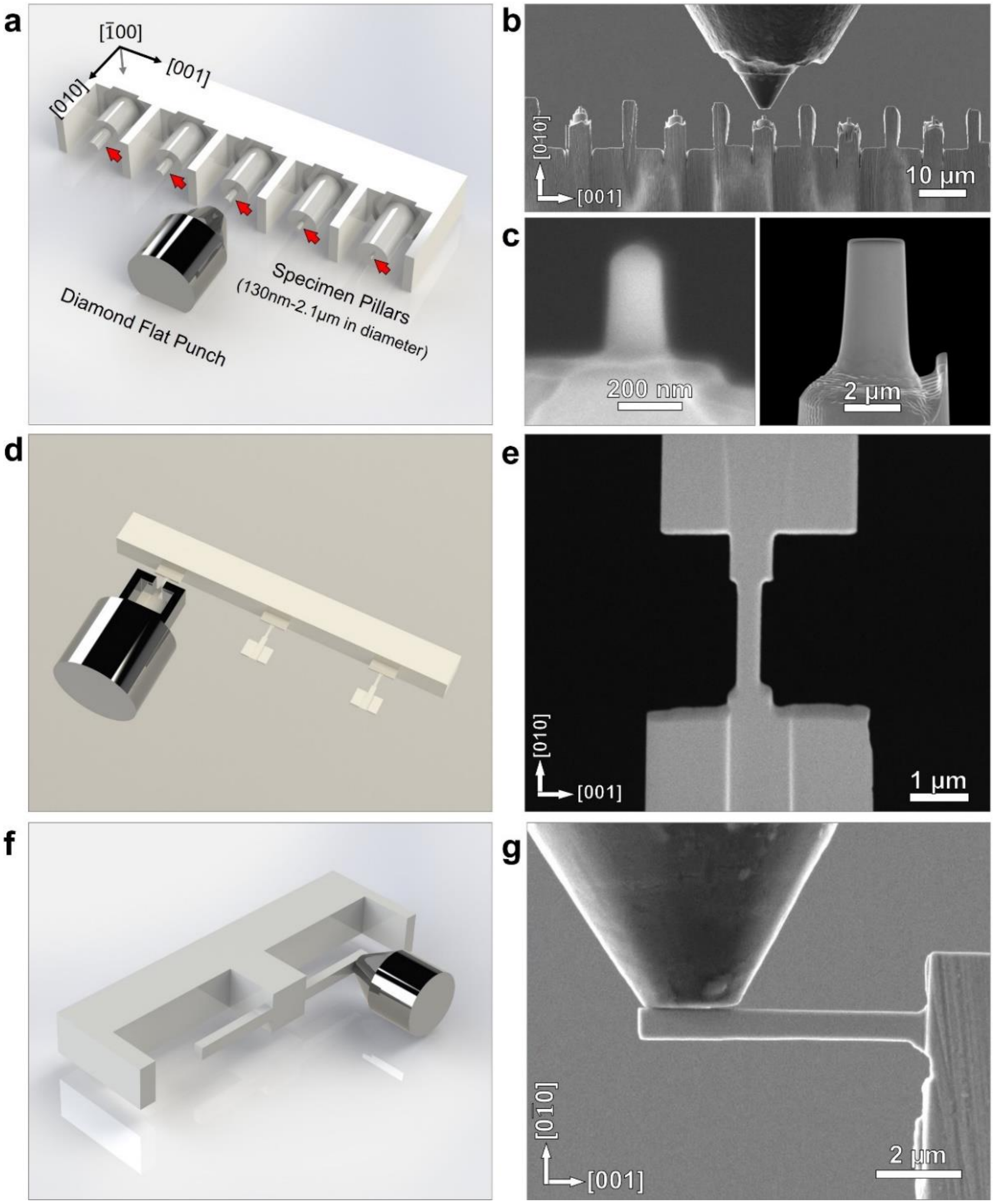

Supplementary Fig. 2. Experimental set up of compression, tensile and bending tests along [010]$_{pc}$ loading direction. a, **d**, **f**, Schematics showing the experimental setup for compression, tensile and bending tests. In image **a**, the sample orientation and diamond punch are labeled. Pillars are indicated by red arrows. **b**, An SEM image showing the diamond punch and pillars. Walls were prepared between the pillars to prevent milling-induced redeposition. **c**, SEM images showing round pillars for compression tests with diameters of 130 nm and 2 μm, respectively. **e**, **g**, SEM images of dog-bone pillar prepared for tensile test and cantilever beam prepared for bending test, respectively. All samples are prepared by a focused ion beam (FIB).

3. Compression test results of PIN-PMN-PT pillars with diameters ranging from 130 nm ~ 270 nm, with a loading direction of $[010]_{pc}$:

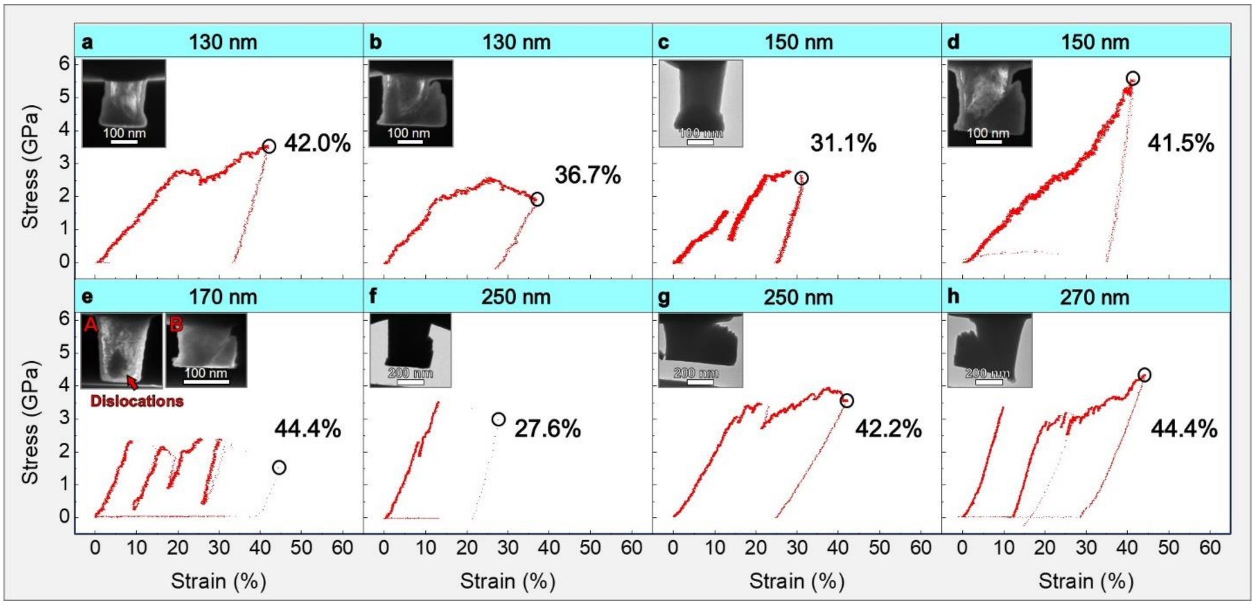

Supplementary Fig. 3. Engineering stress – strain curves obtained during in-situ compression of pillars with diameters ranging from 130 nm ~ 270 nm, and corresponding TEM images of pillars after compression tests. In image **e**, A and B are TEM images showing the pillar before and after the compression test. Dislocations are evident in A as indicated by a red arrow. All stress – strain curves and images are typical of plastic deformation.

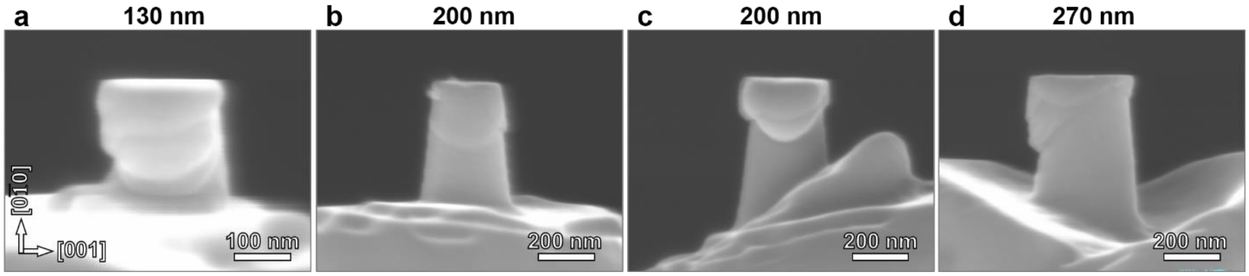

Supplementary Fig. 4. SEM images of compressed PIN-PMN-PT pillars with diameter ranging from 130 ~ 270 nm. Deformation induced slip bands lead to mushroom-shaped pillars.

4. Compression test results of $SrTiO_3$ (STO) single crystal pillars with diameter ranging from 150 nm to 260 nm, and loaded along [010]:

STO pillars were fabricated with 150 nm to 260 nm diameters using the same method as PIN-PMN-PT. Tests were carried out under the same conditions as the PIN-PMN-PT. Results from these experiments are not directly comparable to those previously reported of STO[1, 2], since the stain rate, sample size, and experimental methodology are not the same.

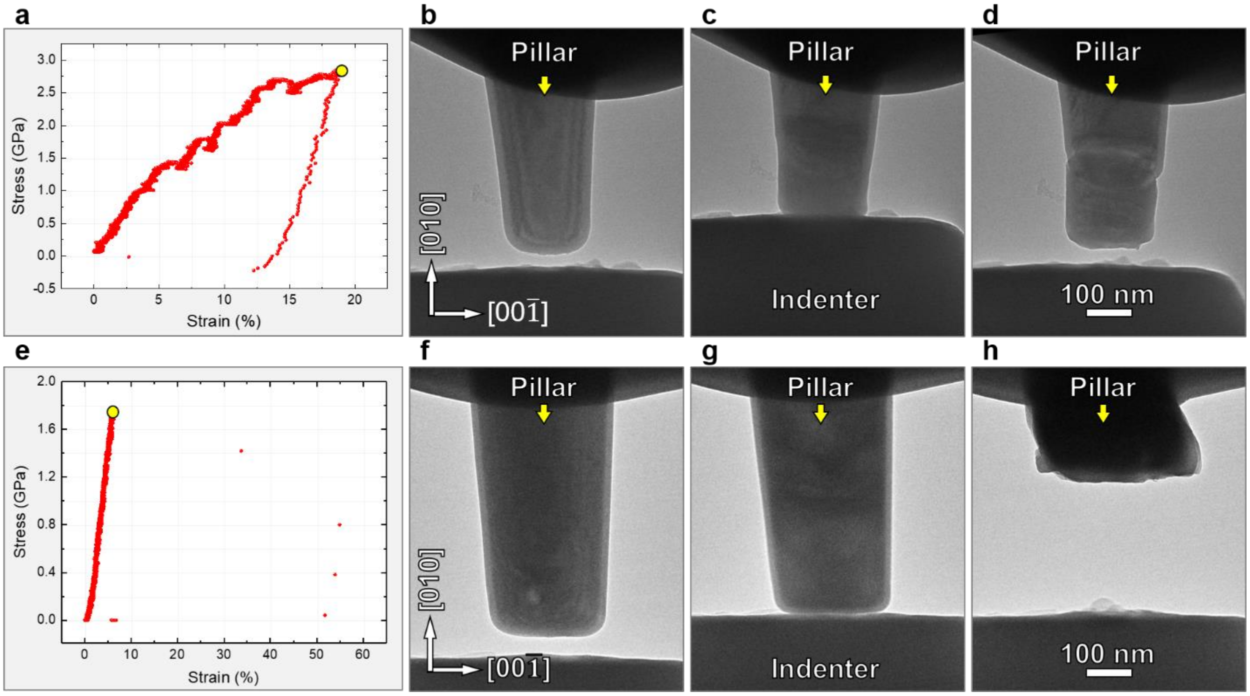

Supplementary Fig. 5. Compression performance tests of STO pillars with diameters of 180 nm (a – d) and 260 nm (e – h). a, **e**, Engineering stress-strain curves recorded during in-situ compression along $[010]_{pc}$. **b**, **f**, TEM images showing morphologies of pillars before compression. **c**, **g**, Snapshots captured from real-time videos at a strain of around 17.8% and 7.5% for 180 nm and 260 nm pillars respectively (corresponding to the maximum load as indicated by yellow circles in **a** and **e**. **d**, **h**, TEM images showing morphologies of pillars after tests. The 180 nm pillar shows some plastic deformation: i.e. the stress – strain curve **a** displays a nonlinear stress plateau, and the TEM image **c** shows a semicircular region of contrast as well as small steps on the side of the pillar. Though some contrast change was identified in image **g** compared to **f**, the 260 nm pillar fails in a brittle way. By comparing Supplementary Figs. 3, 4 and Fig.1**a** – **d** in the main text, it can be identified that the plastic deformation characteristics observed in STO are much less than for PIN-PMN-PT.

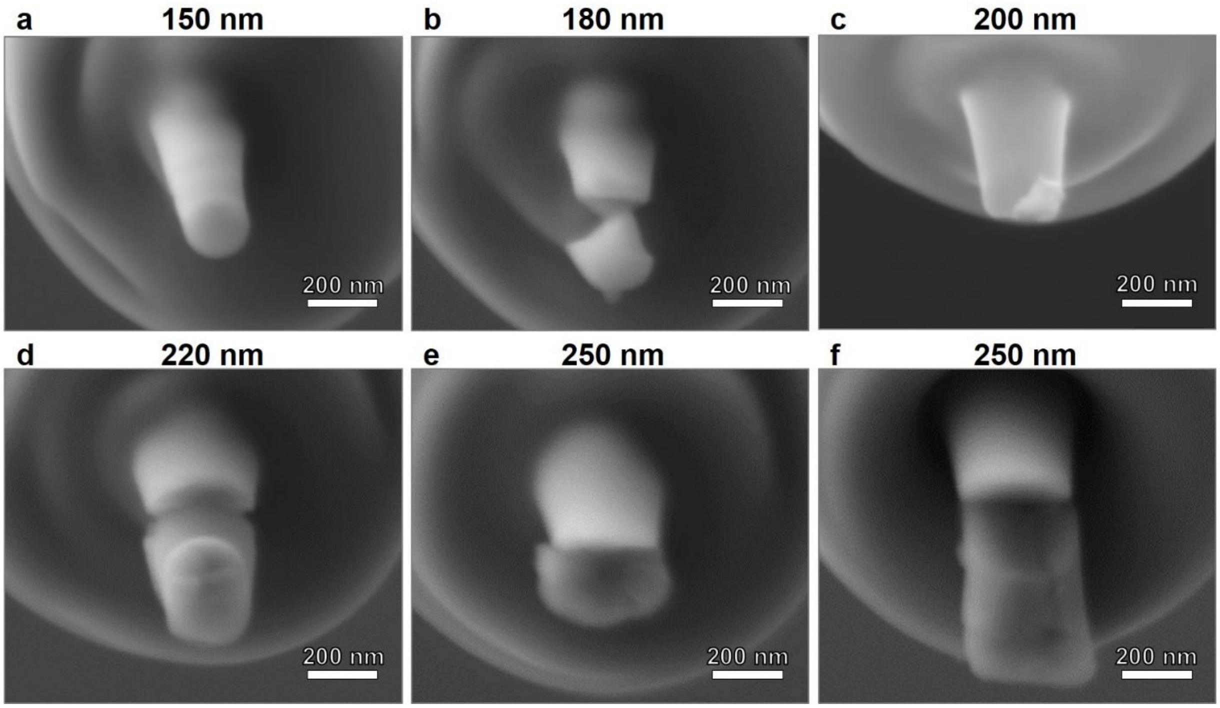

Supplementary Fig. 6. SEM images of compressed $SrTiO_3$ pillars with diameters ranging from 150 nm ~ 250 nm. The loading direction is [010]. Image **a** shows the only pillar that was not fractured, and this sample shows evidence of plastic deformation (slip bands). By comparing Supplementary Fig. 6 and Supplementary Fig. 4, we conclude that PIN-PMN-PT displays much better plasticity than STO.

5. Compression of PIN-PMN-PT pillars with diameters ranging from 500 nm ~ 2.1 μm and loading direction along $[010]_{pc}$:

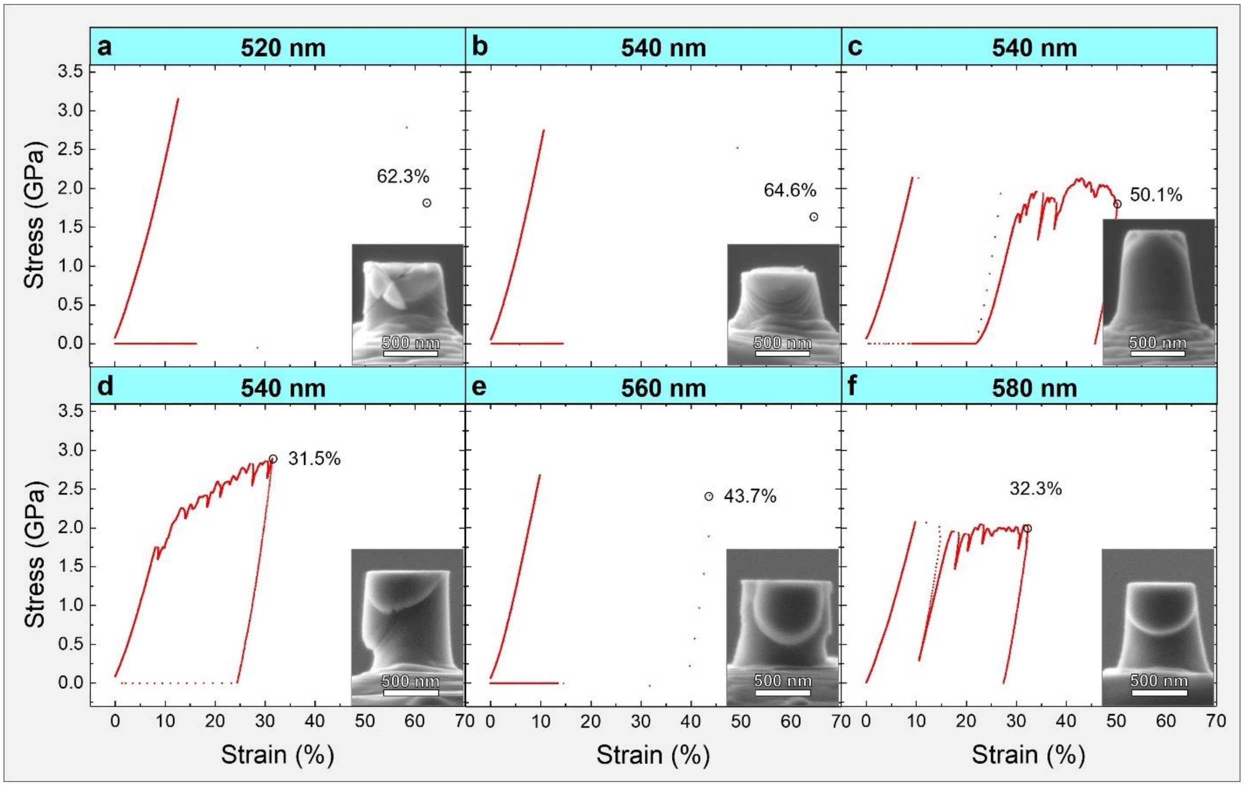

Supplementary Fig. 7. Compression test results of 520 nm ~ 580 nm diameter PIN-PMN-PT pillars. a – **f**, Engineering stress – strain curves. Inset SEM images showing the corresponding morphology of the compressed pillars.

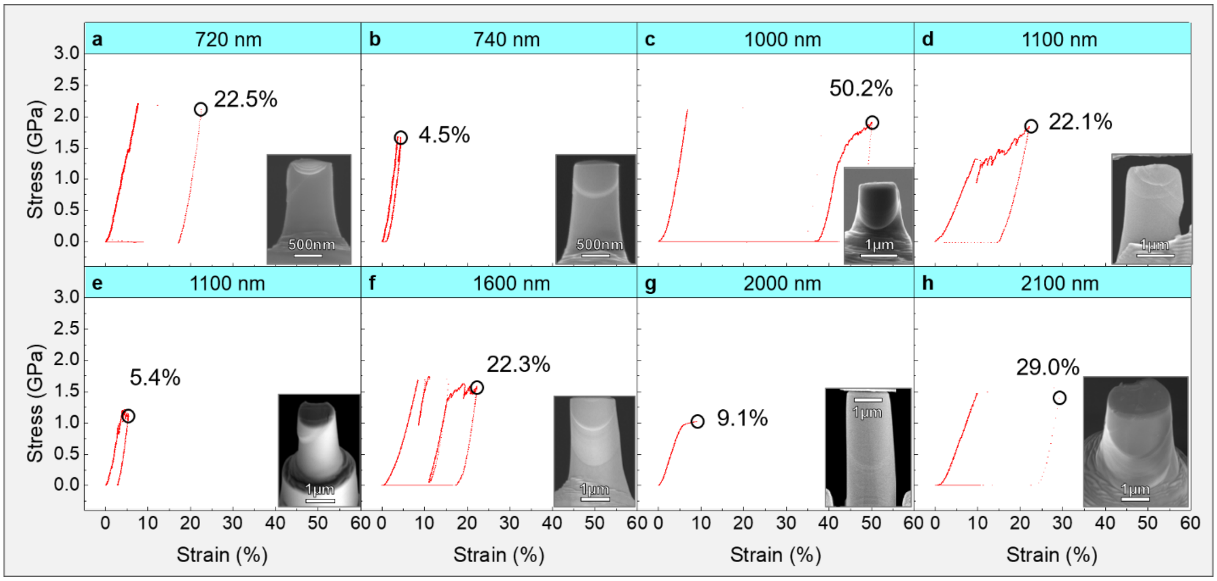

Supplementary Fig. 8. Compression test results of PIN-PMN-PT pillars with diameter ranging from 720 nm ~ 2.1 μm. a – **h**, Engineering stress – strain curves obtained from in-situ compression tests. Insets show SEM images of compressed pillars. {110}<1$\bar{1}$0> slip bands present in every pillar.

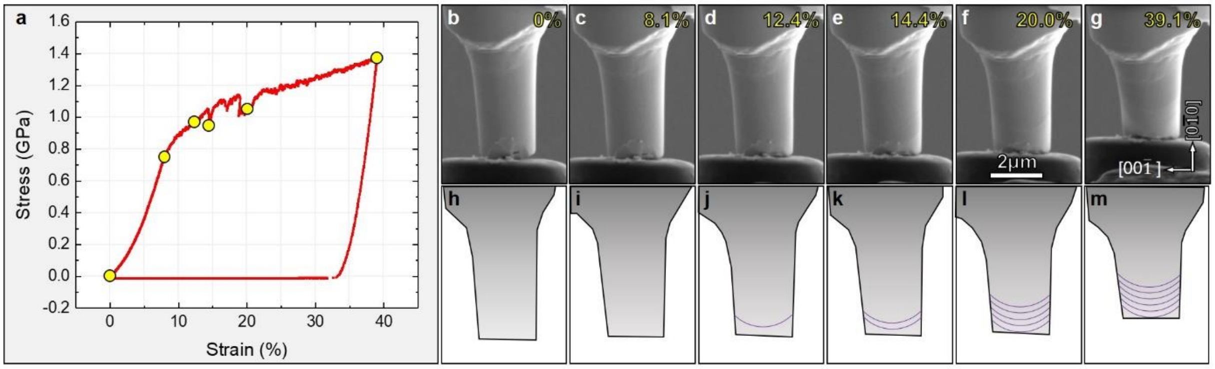

Supplementary Fig. 9. Compression test of a 2.1 μm diameter pillar. a, An engineering stress – strain curve acquired during in-situ compression of the pillar. **b** – **g**, Snapshots from the real time video recording compression process, which correspond to 0, 8.1%, 12.4%, 14.4%, 20.0% and 39.1% strain, respectively. **h** – **m**, Schematics showing the morphology evaluation and slip bands developments corresponding to those in images **b** – **g**. Purple lines indicate slip bands.

6. Inconsistent plasticity in large PIN-PMN-PT pillars:

In Fig. 1i – j, red crosses represent pillars that underwent brittle fracture while black diamonds (open and solid) indicate plastically deformed pillars. In Fig. 1i, it is evident that all pillars with diameters less than 600 nm show excellent plasticity while those with diameters greater than 700 nm show inconsistent plasticity – with some pillars plastically deformed and others not. The fracture strains range from 2.4% ~ 6.2%, comparable to those of elastic strain of 1.3% ~ 6.0% in the plastically deformed pillars (the fracture strain and elastic strain are measured from SEM images of pillars before compression and before fracture / initiation of plastic deformation).

There are a few possible explanations for this phemonemon:

Firstly, pre-existing dislocation sources or dislocations. It is widely accepted that plastic deformation in metals depends highly on the pre-existing dislocation sources.[3, 4] Here, the deformation of PIN-PMN-PT samples is shown to be a result of dislocation slip, and we suppose that the pre-existing dislocations or dislocation sources facilitate plastic deformation, like they do in metals. An example is shown in Supplementary Fig. 3e. Inset B shows a TEM dark-field image of the pillar before compression test, where dislocations are evident, as indicated by a red arrow. Similar to this pillar, a large pillar with diameter of about 2.1 μm, shown in Supplementary Fig. 9, had been nudged prior to the test with a diamond punch, resulting in a crack area (close to the front end as can be seen clearly in Supplementary Fig. 9b). Dislocations are expected in this area, according to the phenomenon observed in Supplementary Fig. 3e. For this pillar, a total strain of 39.1% was obtained, and it is suggested that the excellent plasticity observed in this pillar relates to the pre-introduced dislocations.

Secondly, slight pillar misalignment may facilitate deformation. A perfect alignment along $[010]_{pc}$ means that three slip planes have an equal critical resolved shear stress (CRSS), meaning that multiple slip systems are likely to be operations. Interactions between dislocations on different slip planes may result in crack generation like that observed in Supplementary Fig. 7a and Supplementary Fig. 10.

There are other possible reasons for the inconsistent plasticity instability observed in pillars with diameter greater than 700 nm:

(a) For large pillars, stress concentration tends to occur during loading, especially if the front end of the pillar and the diamond punch do not fit exactly;

(b) Surface effect. Some nanowires show plasticity due to their extremely small size[5], in which surface plays a significant role in determining its performance. In the PIN-MN-PT pillars with diameter greater than 700 nm, the surface effect is significantly reduced, and it is more likely a bulk material in behavior;

(c) In pillars with diameter larger than 700 nm, more slip systems are able to to initiate, resulting in multiple slip bands or dislocation interactions, which easily result in local work hardening and fracture.

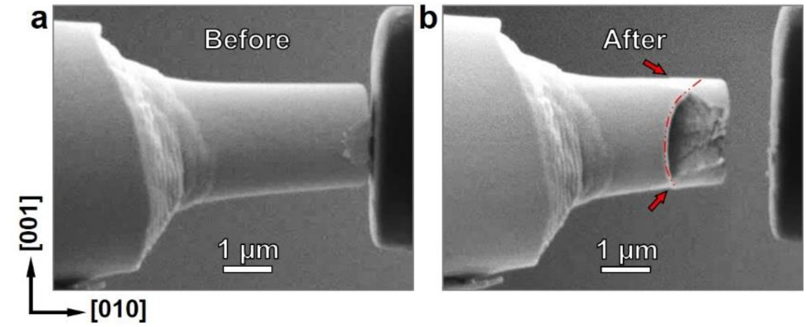

Supplementary Fig. 10. An example showing crack behavior induced by multiple slip system initiation. a, An SEM image of a pillar before compression test. **b**, An SEM image showing the pillar after compression. In image **b**, two red arrows indicate the first and the second slip systems initiated: $(011)[0\bar{1}1]$ and $(01\bar{1})[0\bar{1}\bar{1}]$. The red dashed line indicate the third slip system initiated: $(110)[1\bar{1}0]$. It is clear that crack occured when the third slip band $(110)[1\bar{1}0]$ initiated.

7. Microstructural analysis of compressed pillars:

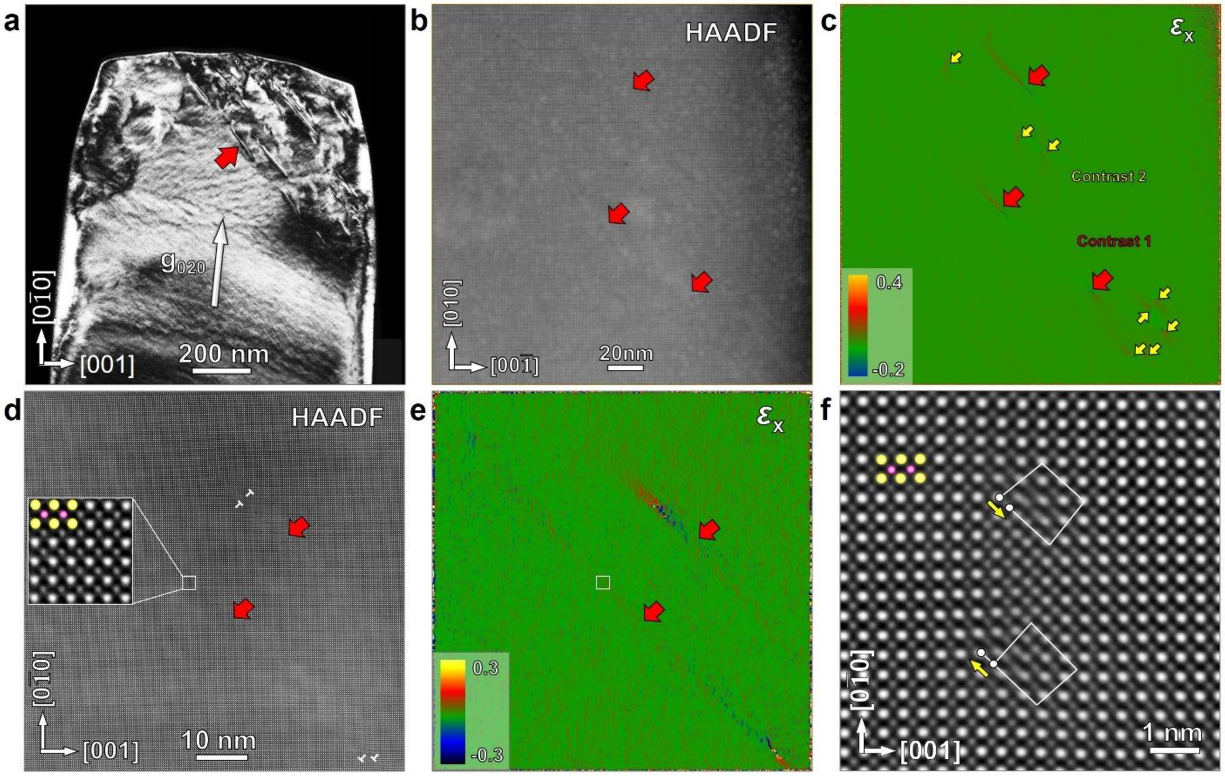

Supplementary Fig. 11. Microstructural investigation of compressed pillars. a, A TEM dark-field image taken using the diffraction vector $\boldsymbol{g}_{020}$, where line contrast can be revealed as indicated by a red arrow. **b** – **c**, A low magnification high-resolution high-angle annular dark-field scanning transmission electron microscopy (HAADF-STEM) image and a corresponding GPA (see methods) of in-plane strain (ε_x, strain along horizontal direction). Line- and spot-like contrast is revealed and indicated by red and yellow arrows respectively. **d** – **e**, A higher magnification HAADF-STEM image and corresponding GPA analysis of in-plane strain (ε_x) of the lines contrast as indicated by red arrows. Inset in **d**, an enlarged HAADF-STEM image of the white square area. Pb: yellow circles; In/Nb/Mg/Ti: pink circles. Higher magnification HAADF-STEM image in **d** reveals that the lines/stripes fall in the wake of pairs of dislocations with Burgers vector of $\frac{1}{2}a$<011> (Supplementary Fig. 12), and are thought to be traces left by the dislocation slip. **f**, A HAADF-STEM image showing a pair of partial dislocations with Burgers vectors of $\frac{1}{2}a[011]$ and $\frac{1}{2}a[0\bar{1}\bar{1}]$ separated by a stacking fault. Yellow arrows indicate the directions of Burgers vectors. White lines trace Burgers circuits.

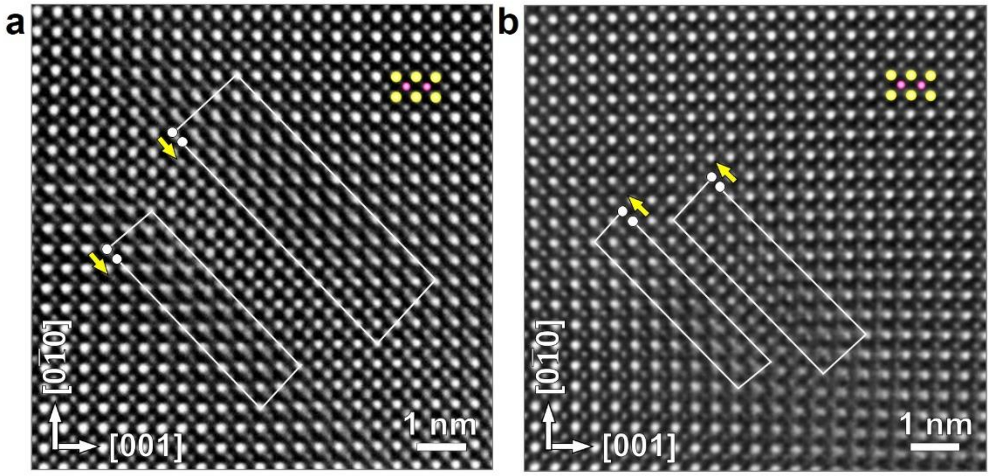

Supplementary Fig. 12. HAADF-STEM images showing dislocations structure in Supplementary Fig. 11d. Images **a** and **b** corresponding to that of upper pair (Burgers vectors of $\frac{1}{2}a[011]$) and lower pair (Burgers vectors of $\frac{1}{2}a[0\bar{1}\bar{1}]$) of dislocations respectively.

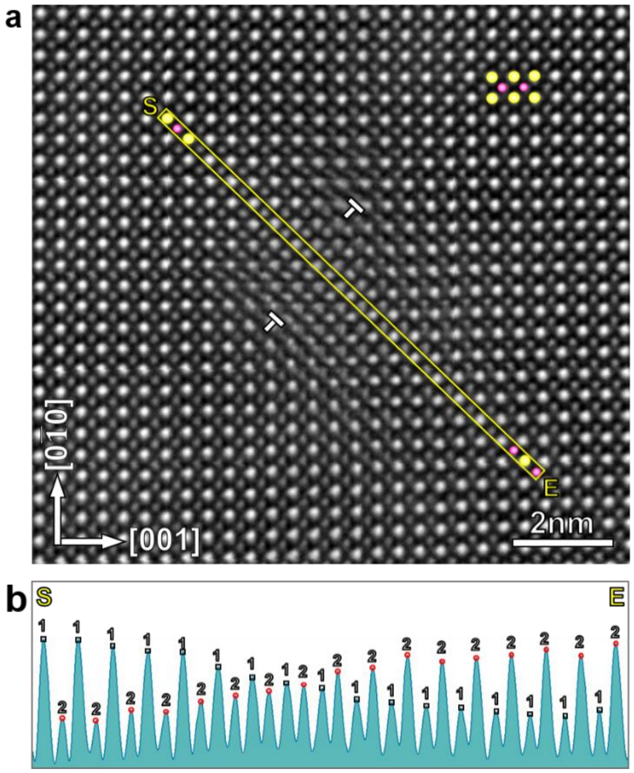

Supplementary Fig. 13. Analysis of antiphase boundary (APB) between a pair of partial dislocations with Burgers vector of *a*[0$\bar{1}\bar{1}$]. **a**, A HAADF-STEM image showing a pair of dislocations with Burgers vectors of $\frac{1}{2}$ *a*[011]. Between two partial dislocations, image spots contrast variation can be identified. At the start (S) and end (E) position, bright (Pb, yellow circles) and weak spots (Ti/In/Mg/Nb, pink circles) arrange in order. However, between S and E, the contrast becomes uniform. **b**, A line profile along S – E in image **a**. In image **b**, Pb and Ti/In/Mg/Nb are labeled as “1” and “2” respectively at the very start position (S). Then “1” and “2” are labeled in sequence until the end position (E). In perfect perovskite lattice, “1” will locate at Pb position and “2” will locate at Ti/In/Mg/Nb position all the time. However, we found that at the end position, “1” locates at Ti/In/Mg/Nb and “2” at Pb positions, suggesting an APB between two partials.

8. Tensile test results of PIN-PMN-PT with loading direction along $[010]_{pc}$:

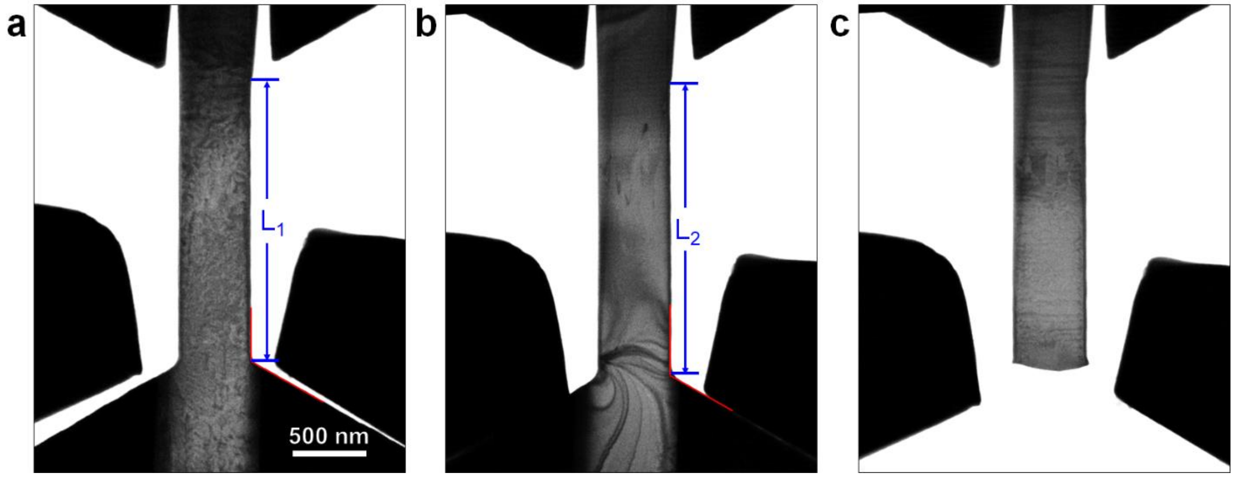

Supplementary Fig. 14. Tensile test of PIN-PMN-PT loaded along $[100]_{pc}$. a, A TEM bright field image showing dog bone sample before tensile test; **b**, the same sample before brittle facture occurs; **c**, A TEM image of the pillar after fracture. The lengths of the pillars were measured in images **a** and **b** as L_1 and L_2 respectively, and an engineering tensile strain is calculated to be around 4.0% using the equation $\frac{L_2-L_1}{L_1} \times 100\%$.

9. Bending test results of PIN-PMN-PT along $[010]_{pc}$:

Flexural strain is calculated using following equation:

$$\varepsilon_f = \frac{3bx}{2l^2} = 8.2\%$$

where b (0.67 μm) and x (1.51 μm) are width and maximum deflection of the cantilever beam, while l (4.32 μm) is the distance between loading point and the base of the cantilever beam. Elastic strain and plastic strain are calculated using the reversible deflection (1.25 μm) and residual deflection (0.26 μm) respectively.

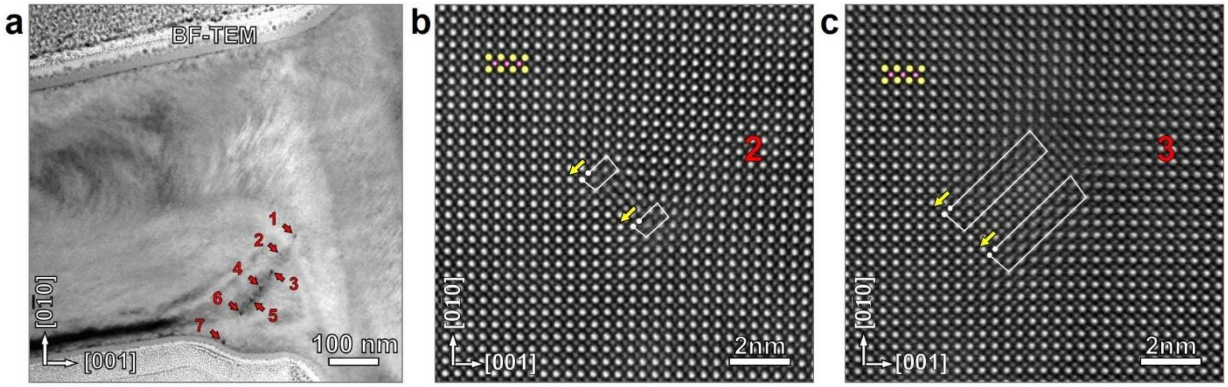

Supplementary Fig. 15. Microstructure analysis of the cantilever beam after bending test. a, A TEM bright image showing dislocation cores contrast labeled by 1 – 7. The image is taken from the root area of the cantilever beam. **b** – **c**, High resolution HAADF-STEM images showing core structure of dislocations #2 and #3.

10. Atomic scale EDS mapping of PIN-PMN-PT:

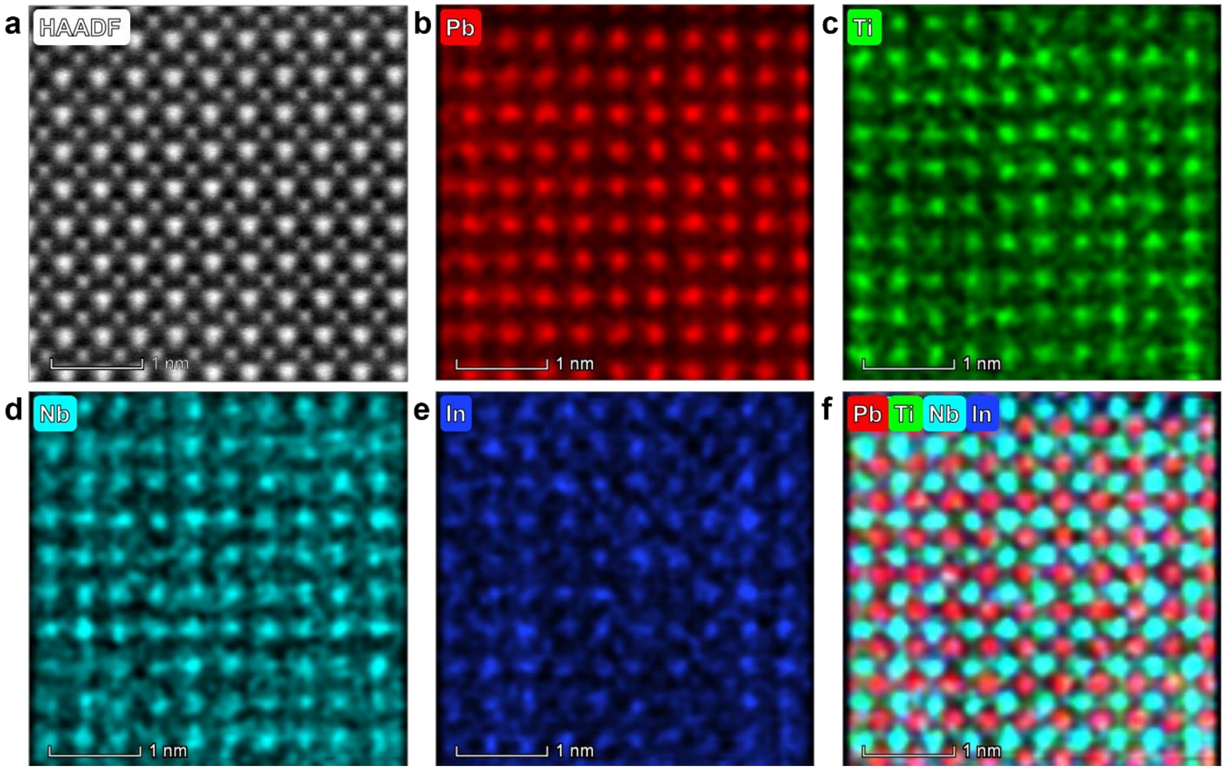

Supplementary Fig. 16. Atomic scale EDS mapping of PIN-PMN-PT acquired. a, A HAADF-STEM image. **b** – **e**, Pb, Ti, Nb and In elemental mappings, respectively. **f**, Color mixing of Pb, Ti, Nb and In element mapping. Uniform distribution of these elements is evident.

11. Three sets of tetragonal subunits.

The perovskite PT-PIN-PMN crystal structure is complex. For instance, even for pure PMN, there is a degree of disordering of Mg^{2+} and Nb^{5+} ions at the B site[6]. For simplicity, in this study, we employed the minimal 5-atom, 10-atom, and 15-atom tetragonal cells for PT ($PbTiO_3$), PIN ($Pb(In_{1/2}Nb_{1/2})O_3$), and PMN ($Pb(Mg_{1/3}Nb_{2/3})O_3$), respectively, as shown in Supplementary Fig. 17. The calculated lattice parameters are tabulated in Supplementary table 1, along with other experimental and theoretical data.

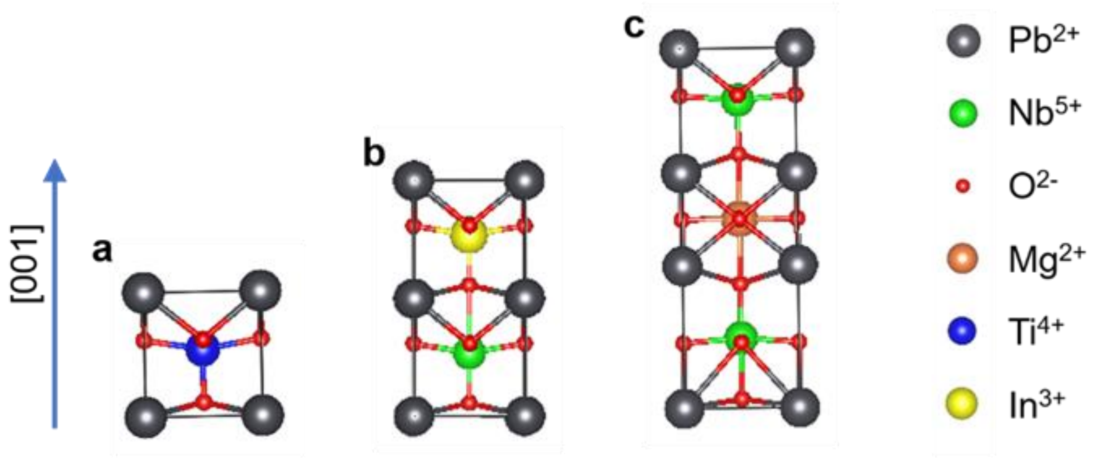

Supplementary Fig. 17. Atomic structures of the three sets of tetragonal subunits. a, PT: $PbTiO_3$, **b**, PIN: $Pb(In_{1/2},Nb_{1/2})O_3$, and **c**, PMN: $Pb(Mg_{1/3}, Nb_{2/3})O_3$.

Supplementary table 1. Calculated lattice parameters (*a* and *c*) of three bulk unit cells along with other theoretical and experimental values.

Materials	*a* (Å)	*c* (Å)	Ref.
PT: ($PbTiO_3$)	3.878	4.499	Present work
	3.846	4.684	DFT[7]
	3.904	4.158	Exp.[8]
PIN: $Pb(In_{1/2}, Nb_{1/2})O_3$	4.006	9.813	Present work
PMN: $Pb(Mg_{1/3}, Nb_{2/3})O_3$	4.015	12.505	Present work
	4.049	-	Exp.[9]

12. **Theoretical microstructural model contains multiple mini-interfaces in the PIN-PMN-PT sample:**

When the three materials (namely PT, PIN and PMN) are mixed at high temperature, the three unit-cells are assumed to retain their chemical identities by considering the electron counting rule such that to maintain the local charge neutrality. Immediately, this leads to a conclusion that the mixing of these three sets of subunits will generate multiple mini-interfaces. Such a scenario is supported by our experimental atomic scale EDS mapping, as shown in Supplementary Fig. 16.

We performed a thorough study on a large number of possible interface structures. Supplementary Fig. 18 shows the relaxed atomic structures for side-by-side and top-down configurations. The corresponding calculated lattice constants are listed in the Supplementary table 2.

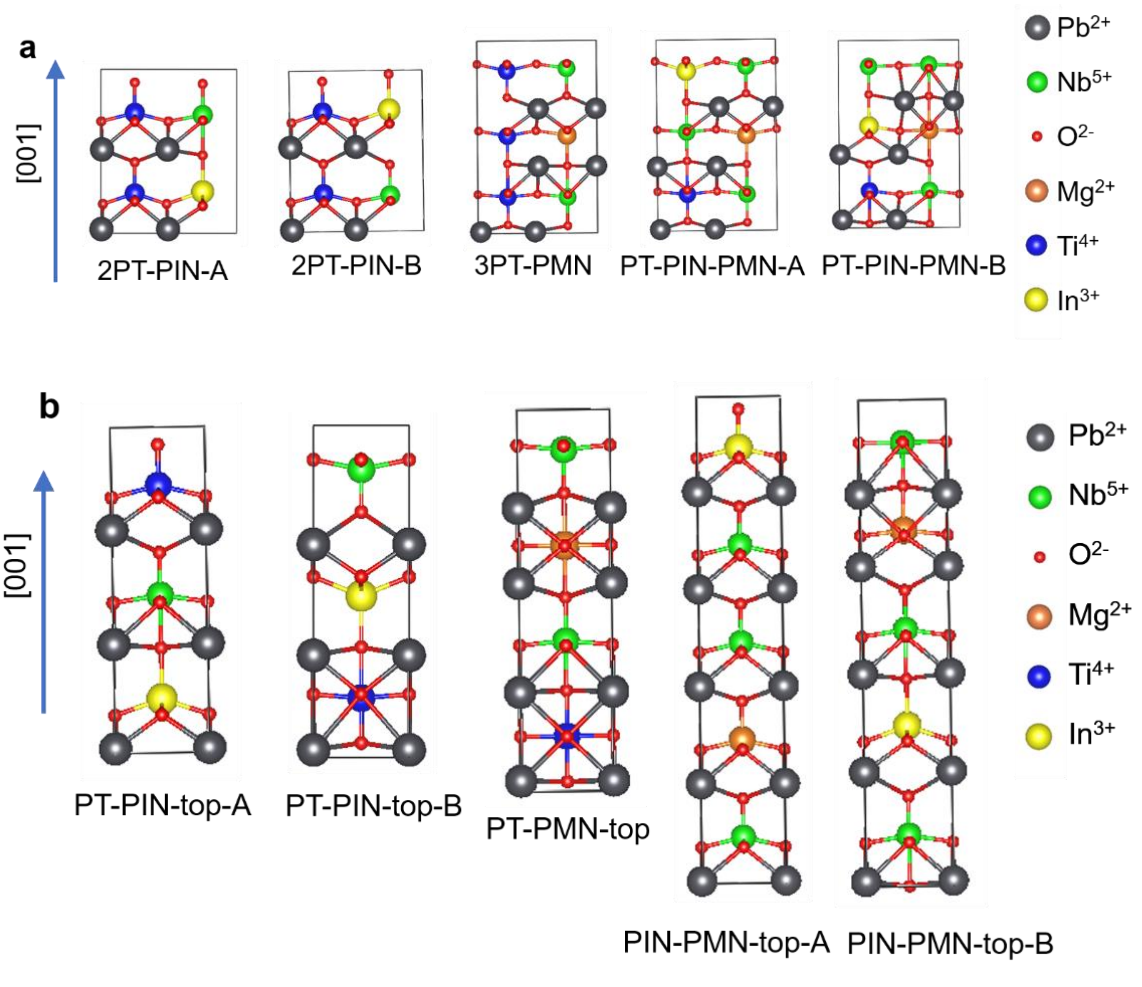

Supplementary Fig. 18. Calculated atomic structures for (a) side-by-side and (b) top-down configurations.

Supplementary table 2: Calculated lattice parameters (a, b and c) and the relative total energies of various interface configurations.

Interface configurations	a (Å)	b (Å)	c (Å)	Relative energy (eV)
side-by side orthorhombic configurations				
2PT-PIN-A	7.840	3.925	9.448	0
2PT-PIN-B	7.839	3.925	9.426	0.061
3PT-PMN	7.915	3.963	12.730	-
PT-PIN-PMN-A	8.045	3.996	12.975	0
PT-PIN-PMN-B	8.108	4.006	12.631	0.641
top-down tetragonal configurations				
PT-PIN-top-A	3.990	3.990	13.754	0.445
PT-PIN-top-B	3.980	3.980	13.764	0
PT-PMN	3.993	3.993	16.442	-
PIN-PMN-top-A	4.002	4.002	22.307	0
PIN-PMN-top-B	4.002	4.002	22.263	0.051
side-by side orthorhombic configurations with $V_O^{\cdot\cdot}$				
2PT-PIN-A- $V_O^{\cdot\cdot}$	7.986	3.832	7.969	
3PT-PMN- $V_O^{\cdot\cdot}$	7.934	3.927	11.714	
PT-PIN-PMN-A- $V_O^{\cdot\cdot}$	8.008	3.957	12.008	
top-down tetragonal configurations with $V_O^{\cdot\cdot}$				
PT-PIN-top-B- $V_O^{\cdot\cdot}$	3.969	3.969	11.448	
PT-PMN-top- $V_O^{\cdot\cdot}$	3.855	3.855	16.277	
PIN-PMN-top-B- $V_O^{\cdot\cdot}$	3.939	3.939	20.702	

It is found that the configurational ordering is found important; for instance, PT-PIN-PMN-B is higher in energy that PT-PIN-PMN by 0.64 eV.

To evaluate the likelihood of interfaces, we calculated the interface formation energies E^f, defined as:

$$E^f\,[(PT)_l(PIN)_m(PMN)_n] = E\_[(PT)_l(PIN)_m(PMN)_n] - l \times E\_[PT] - m \times E\_[PIN] - n \times E\_[PMN],$$

where E is the total energy.

The calculated formation energy values for various interfaces are shown in Supplementary Fig. 19. It is evident that among the side-by-side configurations, the PT-PIN-PMN-A is energetically favorable. For the top-down cases, both PIN-PMN interfaces are favorable – partially due to the small in-plane lattice mismatch between PIN and PMN.

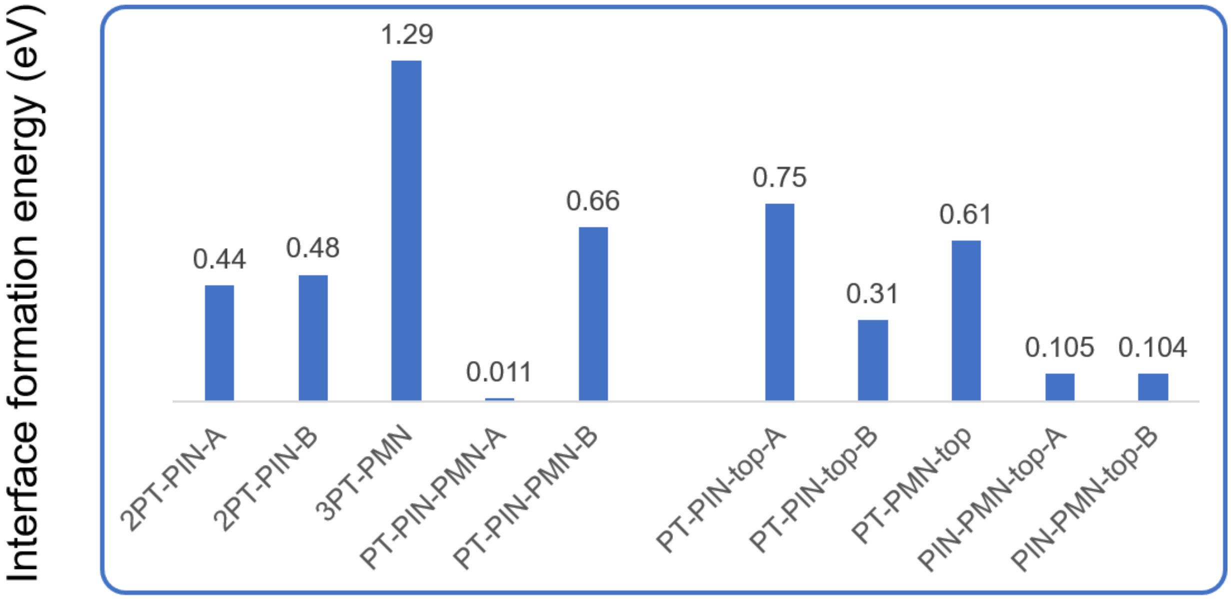

Supplementary Fig. 19. Calculated interface formation energy of various side-by-side and top-down interface configurations.

13. Oxygen vacancies in PIN-PMN-PT:

It is well established that charged vacancies, both oxygen vacancy (V_O) and lead vacancy (V_{Pb}), are the common point defects in $PbTiO_3$ and similar oxides[10, 11]. Here we studied the incorporation of one oxygen vacancy in the identified energetically favorable interfaces. For each interface with one vacancy, we conducted a complete search by calculating different vacancy sites. The relaxed atomic structures are shown in Supplementary Fig. 20.

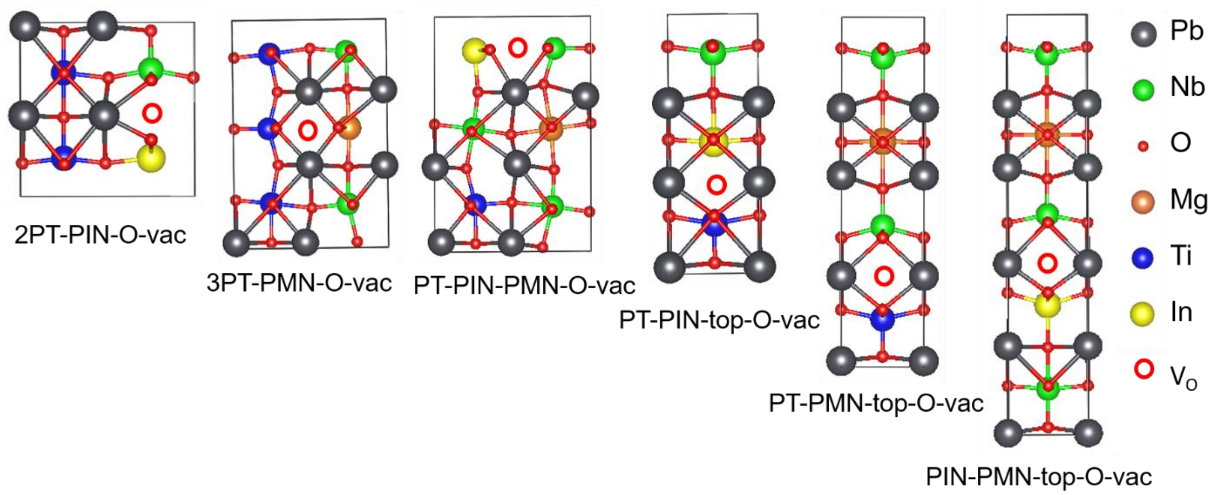

Supplementary Fig. 20. Calculated atomic structure of the interfaces containing O vacancy.

To evaluate the effect of interfaces on the presence of oxygen vacancies, we calculated the V_O formation energy in bulk (PT, PIN and PMN) phases and their interfaces. To make these values comparable, we deliberately used small supercells (10 – 30 atoms) for the bulk phases. However, it is known that a charged vacancies calculation requires corrections to artificial Coulomb interaction, and hence a large supercell. Therefore, in this study, we only focus on neutral vacancies. To evaluate the relative stability of vacancies, we calculated the formation energy by assuming the Pb and O atom reservoirs are bulk Pb and O_2[12]. The calculated neutral V_O and V_{Pb} formation energy in bulk phases and interfaces are shown in Supplementary Fig. 21.

Significantly, the calculated V_O formation energy values in the PIN-PMN-PT interfaces are significantly lower than those in the bulk phases, by ~ 1.5 – 2 eV per V_O. In sharp contrast, the V_{Pb} formation energy values in the interfaces are similar or higher than those in the bulk phases. Thus, our DFT results suggest that, energetically, the presence of interfaces promotes the formation of V_O but not V_{Pb}.

Using Boltzmann distribution[13], the equilibrium oxygen vacancy concentration ratio in interface versus in bulk is:

$$C\text{ (interface)}/C\text{ (bulk)} = \exp\left[(-\Delta E)/kT\right],$$

where ΔE corresponds to the formation energy difference in bulk and in interface, namely ~1.5 – 2 eV, k is the Boltzmann constant and T is the temperature. At T = 300 K, the estimated C ratio is ~ e^{60}-e^{80}! While this value from the simple model may have been severely overestimated considering that (1) the practical process is far from the equilibrium condition, (2) the expected large error-bar of the calculated formation values of neutral vacancies. Nevertheless, our theoretical results clearly demonstrate that in the PT-PIN-PMN sample, the O vacancy concentration is much higher than in the corresponding bulk phases. It is also interesting to notice that for the most configurations shown in Supplementary Fig. 20, the O vacancy prefer to reside at the interface. The calculated lattice constants for the relaxed interfaces containing $V_O^{\cdot\cdot}$ are shown in Supplementary table 2.

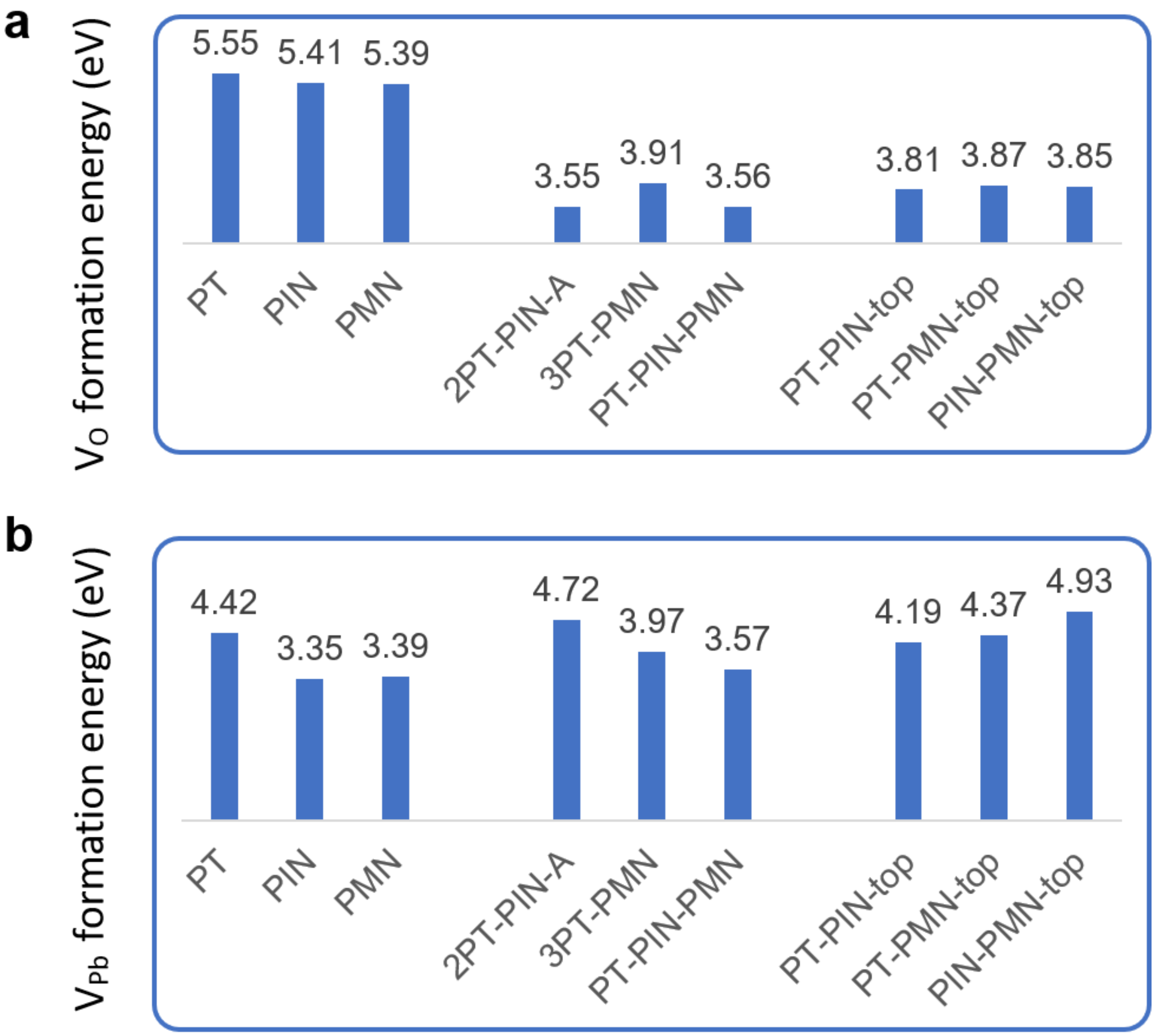

Supplementary Fig. 21. Calculated neutral (a) oxygen vacancy and (b) lead vacancy in bulk (PT, PIN, PMN) and various favorable interfaces.

14. Mechanical properties from first principles:

The mechanical properties can be estimated by elastic moduli such as bulk modulus (B) and shear modulus (G). In particular, the Pugh ratio B/G is conveniently used as an indicator for determining whether a material is ductile or brittle. If the ratio is larger than 1.75, the material tends to be ductile, otherwise brittle. Elastic stiffness constants ($c_{i,j}$) can be obtained by calculating the total energy as a function of appropriate lattice deformation. All the systems studied here are tetragonal structures, each having six independent elastic constant c_{ij}. For the bulk modulus, we used

$$B = \frac{c_{33}c_{12} + c_{11}c_{33} - 2c_{13}^2}{c_{11} + 2c_{33} + c_{12} - 4c_{13}}$$

The calculated bulk moduli for both cubic and tetragonal $PbTiO_3$ agree well with the single crystal experimental data (Ref.42 in the main text). Using DFT-GGA method, the bulk modulus for tetragonal $PbTiO_3$ is 51.2 GPa, compared favourably with other theoretical results (Table 5 in Ref. 42).

For a tetragonal single crystal, the six independent elastic compliances ($S_{i,\,j}$) can be calculated using the calculated $c_{i,j}$ (Ref.43 in the main text) For the anisotropic shear modulus (G) on the (110) plane along <1$\bar{1}$0> direction, one can derive that:

$$G = \frac{1}{2S_{11} - 2S_{12}}$$

where

$$S_{11} = \frac{1}{2}(\frac{c_{33}}{C'} + \frac{1}{c_{11} - c_{12}})$$

$$S_{12} = \frac{1}{2}(\frac{c_{33}}{C'} - \frac{1}{c_{11} - c_{12}})$$

and

$$C' = c_{33}(c_{11}+c_{12}) - 2c_{12}^2$$

The calculated $c_{i,j}$, *B*, *G*, and *B*/*G* values for various bulk phases (PT, PIN and PMN), and interface systems (without and with O vacancy) are summarized in Supplementary table 3.

For the bulk phases, the obtained B/G values are all less than 1.75, in agreement with the fact that these three ceramics are brittle. Similarly, all the pristine interfaces (side-by-side and top-down ones) are also classified brittle. Significantly, introducing charged oxygen vacancy ($V_O^{\cdot\cdot}$) into the interfaces can systematically enhance the B/G values – all are higher than 1.75, with PT-PIN-PMN reaching 2.55 and 3PT-PMN 2.60. Thus, we infer that the high concentration of charge oxygen vacancies induced by interfaces among the three subunit cells is responsible for the unexpected plasticity observed in PIN-PMN-PT.

Supplementary table 3. Calculated elastic constants c_{ij} (kBar), bulk modulus B (GPa), shear modulus G (GPa), and B/G ratio of various bulk and interface systems.

systems	c_{11}	c_{12}	c_{13}	c_{22}	c_{23}	c_{33}	c_{44}	c_{55}	c_{66}	B	G	B/G
PT	2233	901	600	2233	600	519	955	491	491	51.2	66.6	0.77
PIN	1762	664	431	1762	431	525	693	268	268	51.5	54.9	0.94
PMN	3150	839	822	3145	822	1800	898	685	685	135.5	115.5	1.17
2PT-PIN	1926	740	537	2019	549	617	784	374	386	61.0	59.3	1.03
3PT-PMN	2592	802	573	2710	604	791	989	680	360	75.6	89.5	0.84
PT-PIN-PMN	2361	832	828	2668	771	1107	896	617	632	103.2	76.4	1.35
PT-PIN-top	2071	762	462	2071	462	618	788	382	382	59.6	65.5	0.91
PT-PMN-top	3217	939	902	3217	902	2096	929	750	750	149.5	113.9	1.31
PIN-PMN-top	2452	773	540	2470	540	754	819	406	406	71.9	83.9	0.86
2PT-PIN-$V_O^{\cdot\cdot}$	3076	1349	1127	3183	1215	1780	883	602	695	153.4	86.4	1.78
3PT-PMN-$V_O^{\cdot\cdot}$	1935	967	738	2899	1193	2648	776	1039	759	125.7	48.4	2.60
PT-PIN-PMN- $V_O^{\cdot\cdot}$	2168	1067	904	3133	1215	2646	744	917	729	141.0	55.0	2.56
PT-PMN-top- $V_O^{\cdot\cdot}$	3044	1317	1039	3044	1039	2076	1121	679	679	158.2	86.3	1.83
PT-PIN-top-$V_O^{\cdot\cdot}$	2060	853	620	2860	620	2019	996	578	578	114.4	60.3	1.90
PIN-PMN-top- $V_O^{\cdot\cdot}$	2820	1350	1039	2810	1037	1909	951	526	526	151.4	735	2.06

15. Electronic structure: bonding analysis using charge density plots

From a fundamental perspective, material ductility and plasticity are intrinsically associated to the atomic bonding. For instance, metallic materials normally have an appreciable ductility because the valence electrons in metals are not localized and the metallic bonds are not directional – facilitating dislocation slip.

To understand the high-density $V_O^{\cdot\cdot}$-induced brittle-to-ductile transition in PIN-PMN-PT, we conducted a systematic bonding analysis by calculating the charge density of various interfaces containing an oxygen vacancy. Supplementary Figs. 22 a – b show the (020) planes (containing B-site atoms and O atoms) of the favorable side-by-side and top-down interfaces each containing one $V_O^{\cdot\cdot}$. In general, in PIN-PMN-PT, the covalency in Mg-O, In-O and Ti-O bonds is weaker than that in Nb-O. This is evident from the 2D charge density plots by treating Pb-5d, Nb-4p, Mg-2p, Ti-3p and In-4d semi-core states as valence states. Another factor in favoring the $V_O^{\cdot\cdot}$ site is the aforementioned interface effect. For all these cases, it is evident that the presence of a $V_O^{\cdot\cdot}$ (removing one O atom and also two electrons) eliminates the local covalent bonding, facilitating dislocation slip.

To compare with the effect of V_{Pb}'' (removing one Pb atom but adding two electrons), taking PT-PIN-PMN interface as an example, Supplementary Fig. 22 (c) shows the 2D charge density plots of the pristine interface and that containing one $V_O^{\cdot\cdot}$ on (020) plane, and of the pristine interface and that containing one V_{Pb}'' on (010] plane (containing only Pb atoms and O atoms). The incorporation of a V_{Pb}'' results in strong structural distortion. While it eliminates the local covalent bonding (as $V_O^{\cdot\cdot}$ does), it also enhances some of the other Pb-O-Pb covalent bonding as highlighted in Supplementary Fig. 22 (c). Overall, lead vacancies V_{Pb}'' would deteriorate the ductility and plasticity. Such an effect can be attributed to the added (instead of removed) electrons for bonding enhancement and structural relaxation.

a

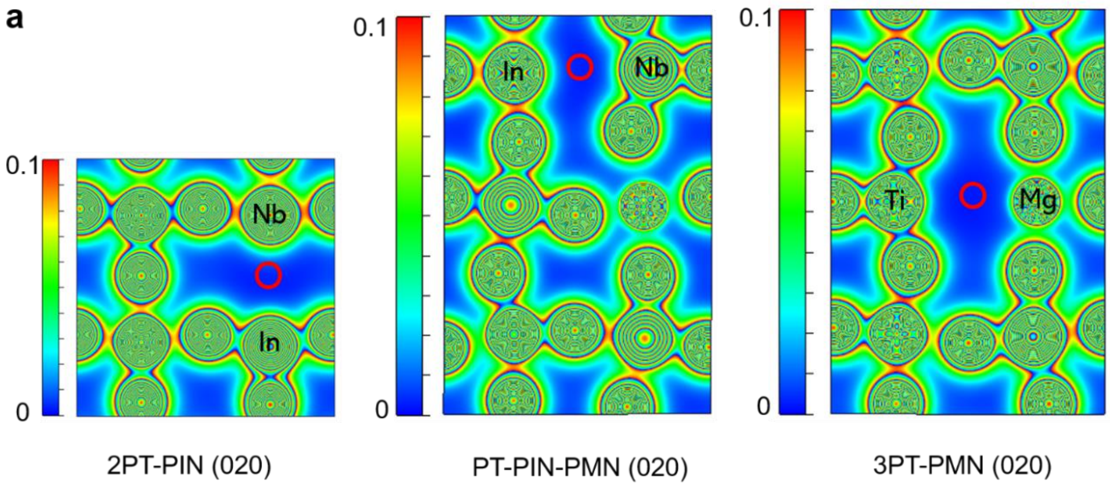
0.1
0
Nb
In
2PT-PIN (020)
In
Nb
PT-PIN-PMN (020)
Ti
Mg
3PT-PMN (020)

b

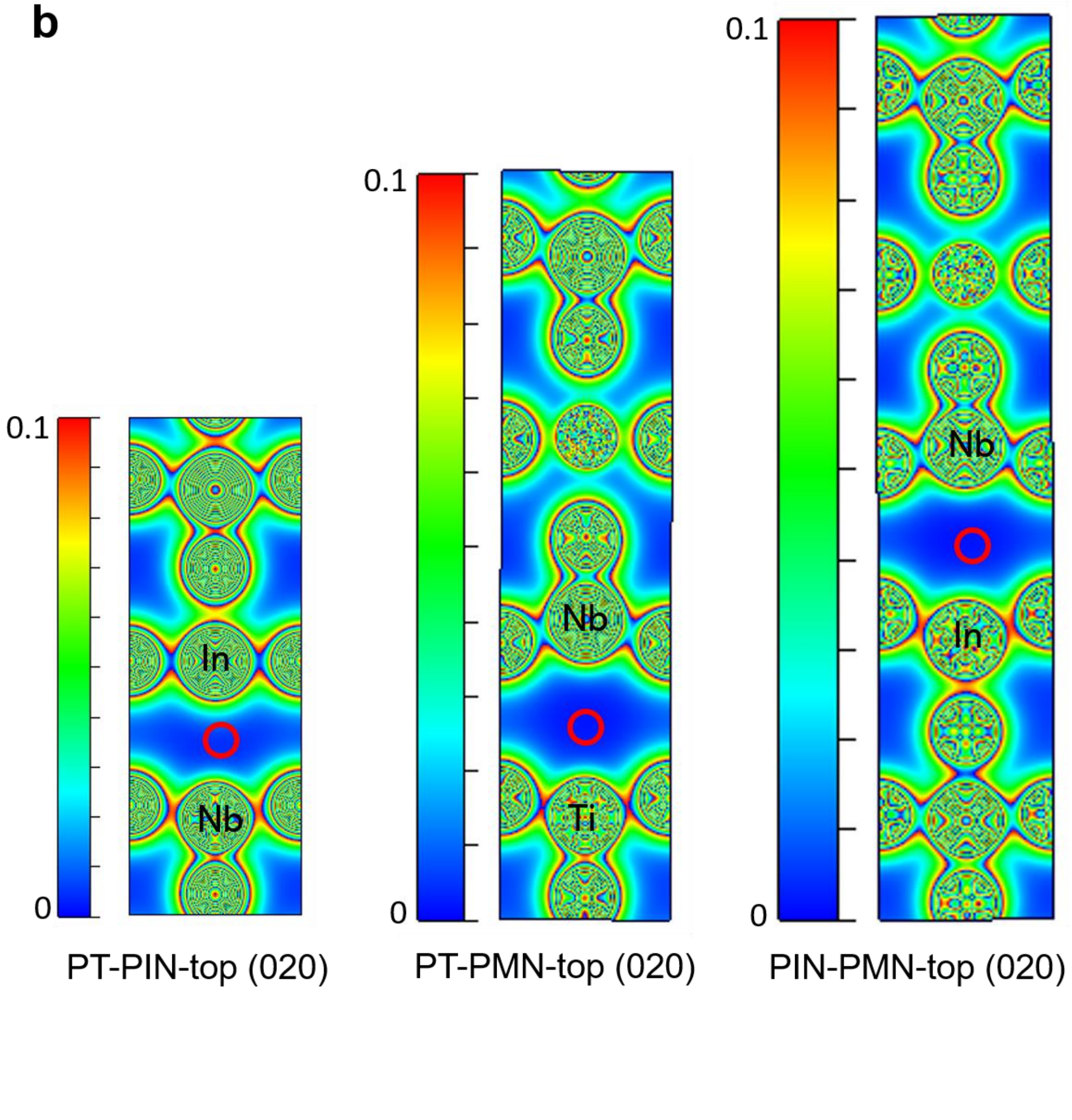
0.1
0
In
Nb
PT-PIN-top (020)
Nb
Ti
PT-PMN-top (020)
Nb
In
PIN-PMN-top (020)

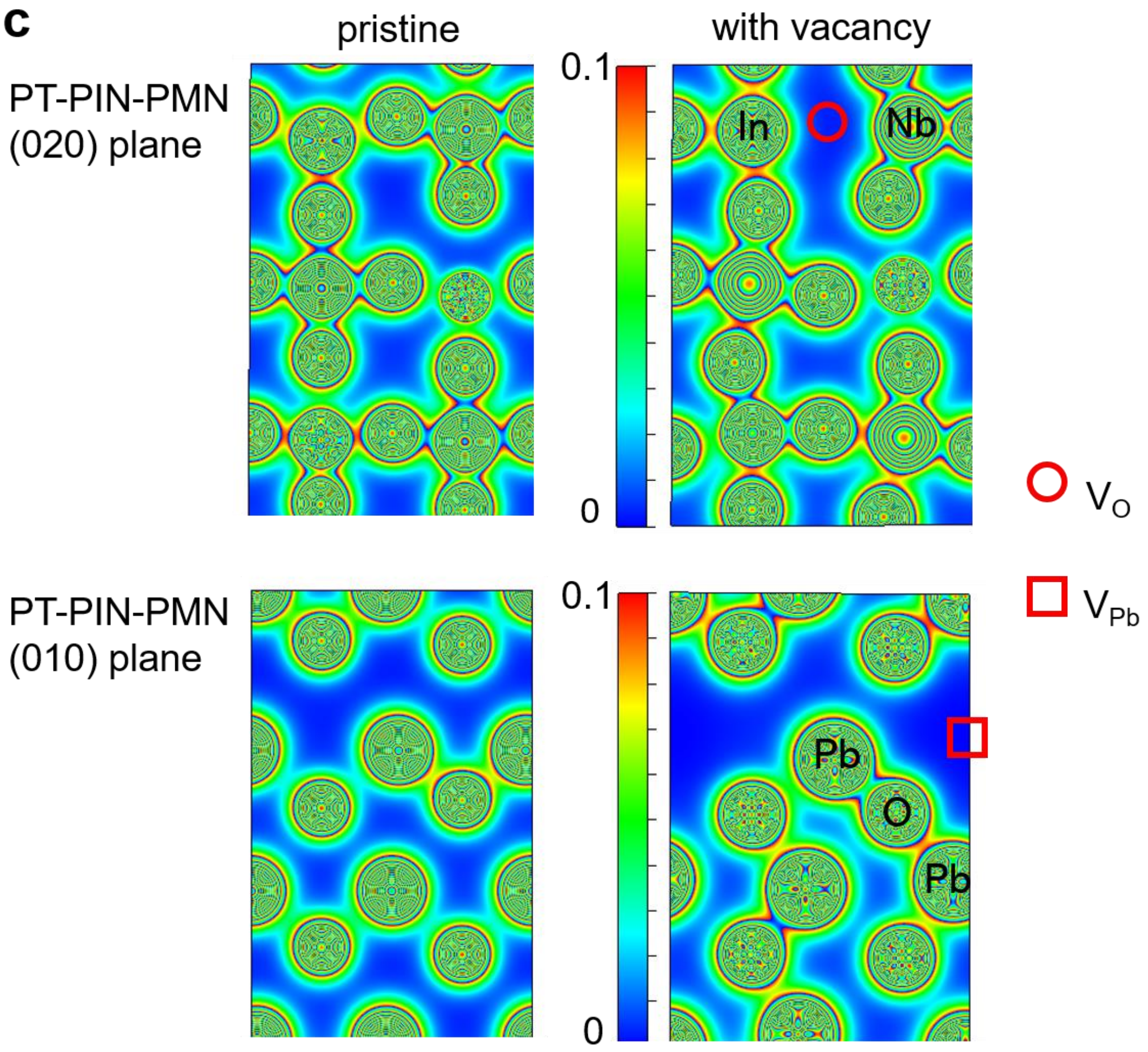

Supplementary Fig. 22. Calculated charge density contour plots (assign colors recursively). a and **b** show the (020) planes of the favorable side-by-side and top-down interfaces each containing one $V_O^{\cdot\cdot}$, respectively. **c** shows the PT-PIN-PMN (020) plane with and without one $V_O^{\cdot\cdot}$ (upper panel), compared with the (010) plane with and without one V_{Pb}'' (lower panel).

16. Comparison of the mechanical test results of Mn-doped ($V_O^{\cdot\cdot}$-rich) and Sm-doped (V_{Pb}''-rich) PIN-PMN-PT:

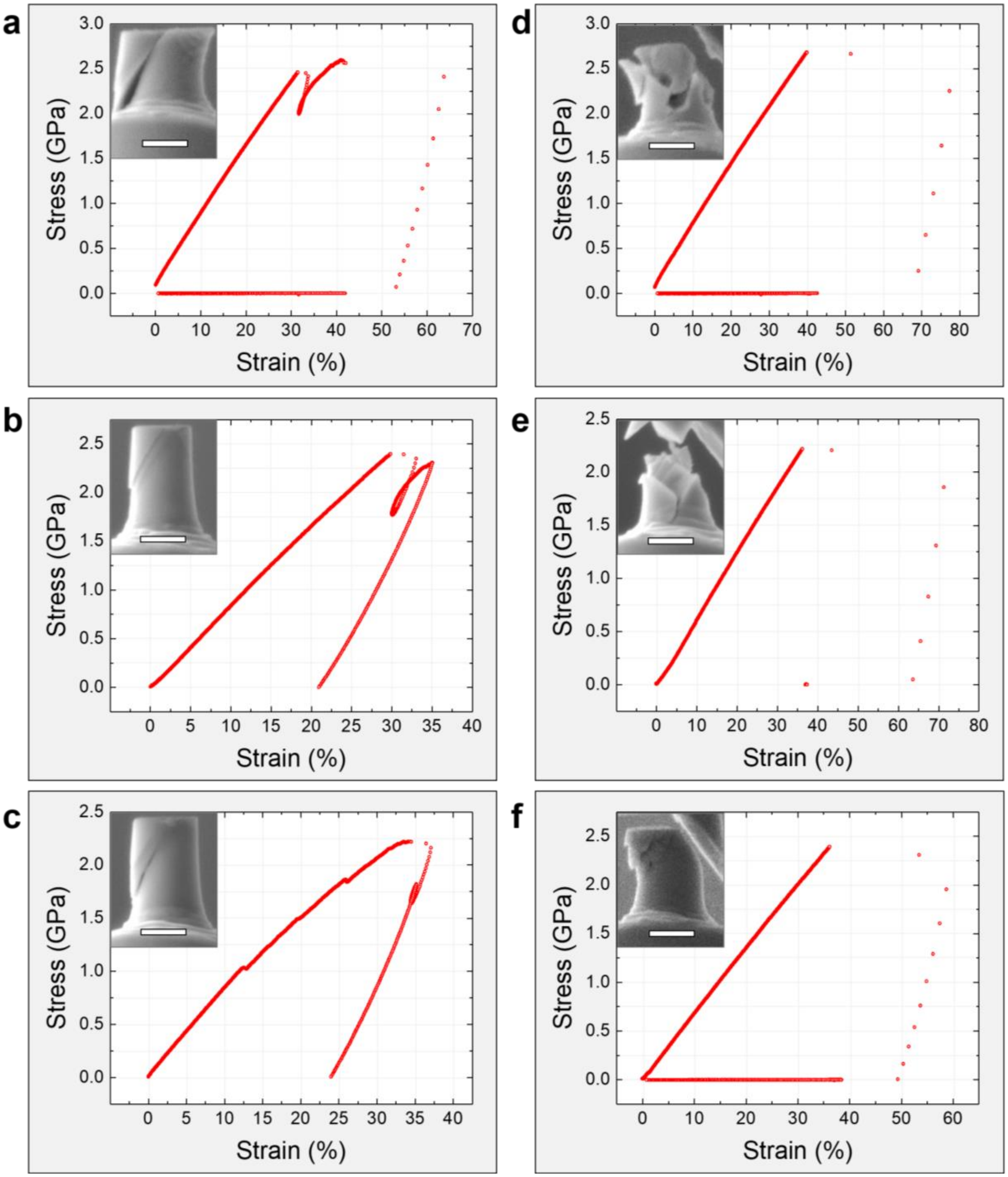

Supplementary Fig. 23. Compression tests results of Sm-doped PIN-PMN-PT with the loading direction along [010]$_{pc}$. a – f shows six engineering strain-stress curves (as obtained). The SEM images of the compressed pillars are shown in the corresponding insets. Three pillars are plastically deformed (**a** – **c**) while the other three ones brittle fractured (**d** – **f**). scale bar: 500 nm.

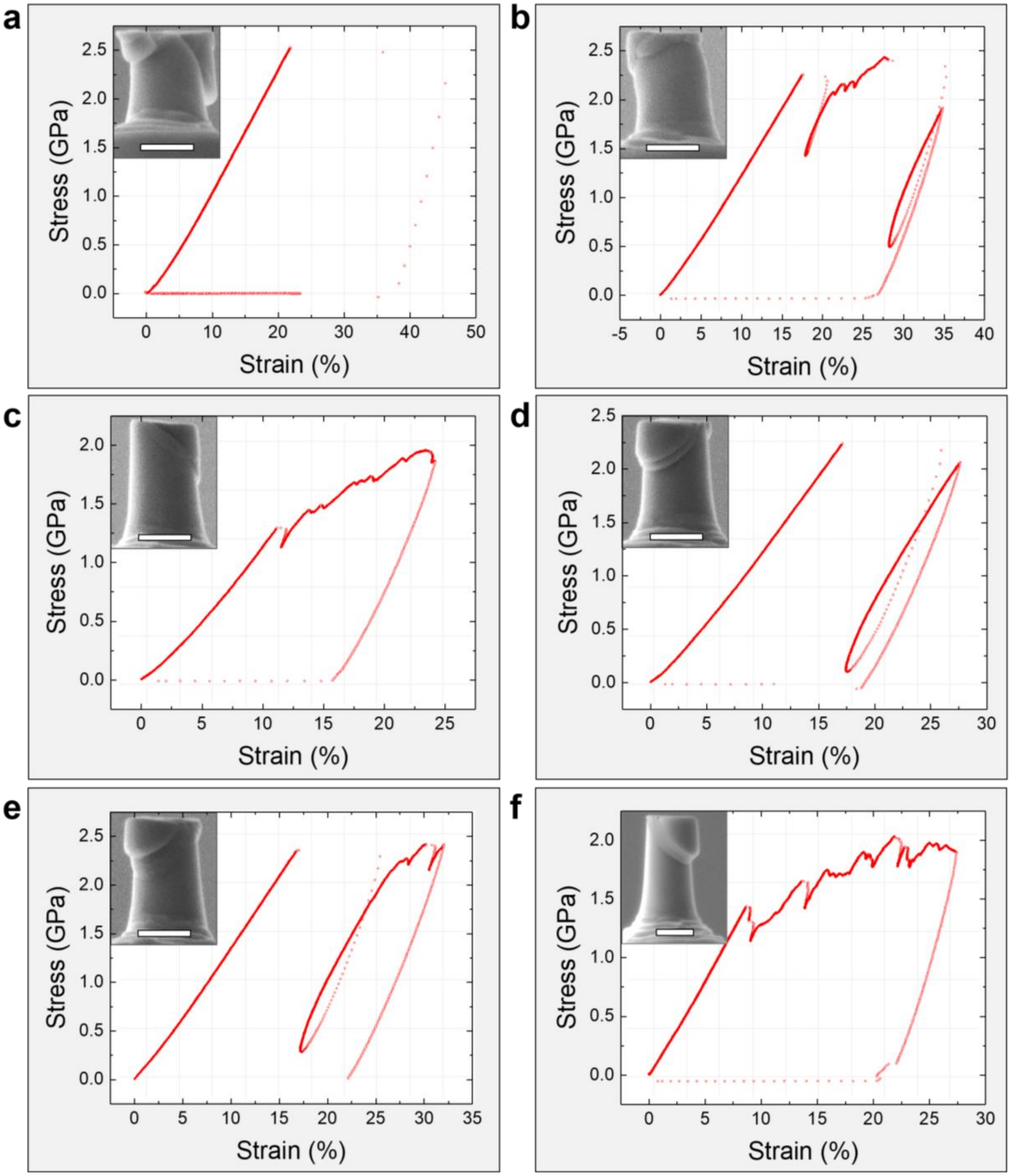

Supplementary Fig. 24. Compression test of Mn-doped PIN-PMN-PT with a loading direction of $[010]_{pc}$. Six pillars were compressed and the corresponding engineering stain-stress curves (as obtained) and SEM images of compressed pillars are shown in **a** – **f**. Scale bar: 500 nm.

References:

1. Brunner, D., Taeri-Baghbadrani, S., Sigle, W. & Ruhle, M. Surprising results of a study on the plasticity in strontium titanate. *J. Am. Ceram. Soc.* **84**, 1161–1163 (2001).
2. Gumbsch, P., Taeri-Baghbadrani, S., Brunner, D., Sigle, W. & Ruhle, A. Plasticity and an inverse brittle-to-ductile transition in strontium titanate. *Phys. Rev. Lett.* **87**, 085505 (2011)
3. Tang, H., Schwarz, K. W. & Espinosa, H. D. Dislocation-source shutdown and the plastic behavior of single-crystal micropillars. *Phys. Rev. Lett.* **100**, 185503 (2008)
4. Cui, Y. N., Lin, P., Liu, Z. L. & Zhuang, Z. Theoretical and numerical investigations of single arm dislocation source controlled plastic flow in FCC micropillars. *Int. J. Plasticity*, **55,** 279–292 (2014).
5. Wang, Y. B., et al. Super Deformability and Young's Modulus of GaAs Nanowires. *Adv. Mater.*, **23**, 1356–1360 (2011).
6. Suewattana, M. & Singh, D. J. Electronic structure and lattice distortions in $PbMg_{1/3}Nb_{2/3}O_3$ studied with density functional theory using the linearized augmented plane-wave method. *Phys. Rev. B* **73**, 224105 (2006).
7. Weston, L., Cui, X. Y., Ringer, S. P. & Stampfl, C. Bistable Magnetism and Potential for Voltage-Induced Spin Crossover in Dilute Magnetic Ferroelectrics. *Phys. Rev. Lett.* **114**, 247601 (2015).
8. Kuroiwa, Y., et al. Evidence for Pb-O covalency in tetragonal $PbTiO_3$. *Phys. Rev. Lett.* **87**, 217601 (2001).
9. Bonneau, P., et al. X-Ray and Neutron-Diffraction Studies of the Diffuse Phase-Transition in $PbMg_{1/3}Nb_{2/3}O_3$ Ceramics. *J. Solid State Chem.* **91**, 350–361 (1991).
10. Yao, Y. P. & Fu, H. X., Charged vacancies in ferroelectric $PbTiO_3$: Formation energies, optimal Fermi region, and influence on local polarization. *Phys. Rev. B* **84**, (2011).
11. Shimada, T., Ueda, T., Wang, J. & Kitamura, T., Hybrid Hartree-Fock density functional study of charged point defects in ferroelectric $PbTiO_3$. *Phys. Rev. B* **87**, (2013).

12. Cui, X. Y., et al. Role of embedded clustering in dilute magnetic semiconductors: Cr doped GaN. *Phys. Rev. Lett.* **95**, 256404 (2005).

13. Osorio-Guillen, J., Lany, S., Barabash, S. V. & Zunger, A. Nonstoichiometry as a source of magnetism in otherwise nonmagnetic oxides: Magnetically interacting cation vacancies and their percolation. *Phys. Rev. B* **75**, 184421 (2007).
